# An Evaluation of Traits, Nutritional, and Medicinal Component Quality of *Polygonatum cyrtonema* Hua and *P. sibiricum* Red.

**DOI:** 10.3389/fpls.2022.891775

**Published:** 2022-04-18

**Authors:** Yan Hu, Minzhen Yin, Yunjun Bai, Shanshan Chu, Ling Zhang, Mei Yang, Xiaowen Zheng, Zhengyang Yang, Junling Liu, Lei Li, Luqi Huang, Huasheng Peng

**Affiliations:** ^1^School of Pharmacy, Anhui University of Chinese Medicine, Hefei, China; ^2^National Resource Center for Chinese Materia Medica, China Academy of Chinese Medical Sciences, Beijing, China; ^3^Research Unit of DAO-DI Herbs, Chinese Academy of Medical Sciences, Beijing, 2019RU57, China; ^4^Anhui Provincial Institute for Food and Drug Control, Hefei, China; ^5^Jinzhai Senfeng Agricultural Technology Development Co., Ltd., Lu’an, China

**Keywords:** Polygonati rhizoma, different types, nutritional and medicinal qualities, multivariate statistical analysis, quality evaluation

## Abstract

Polygonati rhizoma (*Huangjing* in Chinese) is a traditional and classic dual-purpose material used in food and medicine. Herbalists in China and Japan have noticed several different rhizome types in *Huangjing* with different qualities. Rhizome of *Polygonatum cyrtonema* Hua and *P. sibiricum* Red. is divided into five types: “*Jitou*-type” Polygonati rhizoma (JTPR), atypical “*Jitou*-type” Polygonati rhizoma (AJTPR), “*Jiang*-type” Polygonati rhizoma (JPR), “Cylinder-type” Polygonati rhizoma (CPR), and “*Baiji*-type” Polygonati rhizoma (BJPR). This study observed the microstructure and histochemical localization of polysaccharides, saponins, and proteins in *Huangjing*. Nutritional and medicinal component data and antioxidant capacity (DPPH and ABTS) were analyzed to evaluate the quality of different types of *Huangjing*. The results showed that the comprehensive quality of the rhizomes, BJPR and JTPR, was better, regardless of their nutritional or medicinal values. Altogether, these results could recommend future breeding efforts to produce *Huangjing* with improved nutritional and medicinal qualities.

## Introduction

The genus *Polygonatum* (Liliaceae) comprises more than 71 species that are widely distributed in China, Japan, Korea, India, Russia, Europe, and North America. More than half of these species have been used in medicine and food (Zhao et al., 2019). Polygonati rhizoma, commonly known as *Huangjing* in China, is the dried rhizome of *Polygonatum sibiricum* Red., *P. kingianum* Coll. et Hemsl., and *Polygonatum cyrtonema* Hua (The State Pharmacopoeia Committee of China, 2020). In traditional Chinese medicine clinical practice, *Huangjing* has multiple effects: invigorating Qi and nourishing Yin, moistening the lungs, invigorating the spleen, and benefiting the kidneys. Modern pharmaceutical research has shown that *Huangjing* has been used for the treatment of delaying aging, enhancing immunity, anti-fatigue effects, lowering blood sugar, anti-tumor effects, protecting the cardiovascular system, and improving memory (Zhang et al., 2018b; Zhao et al., 2018b; Li et al., 2020, 2021b) and has enormous potential in the treatment of COVID-19 (Mu et al., 2021). From ancient times, *Huangjing* has also been used as food, such as several well-received sweetmeats, functional beverages, and fruit wine (Li et al., 2021c).

**Figure 1:**
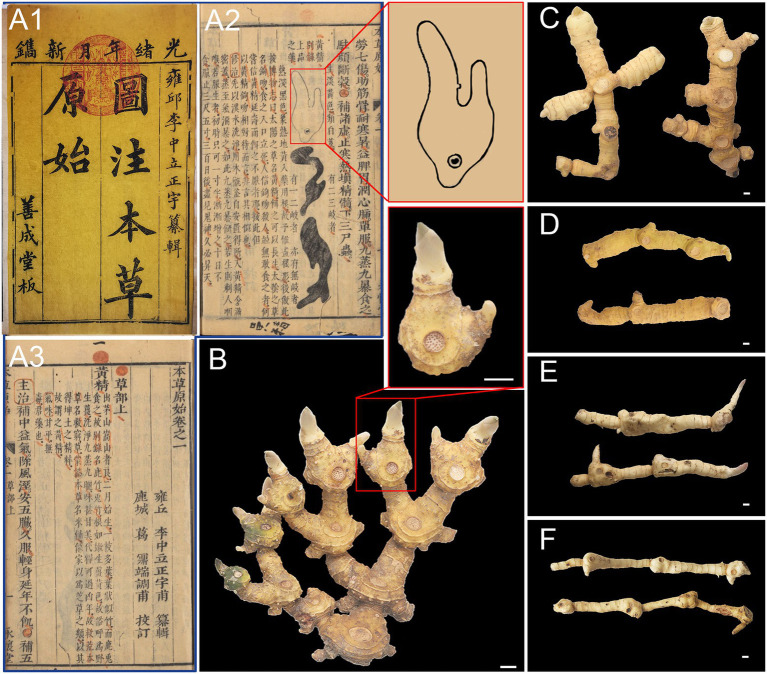
*Huangjing* recorded in *Ben Cao Yuan Shi* 本草原始 **(A1**-**A3)** and five types of *Huangjing* **(B**-**F)**. **(A1)** Shan Cheng Tang Edition, **(A2**,**A3)** Yong Huai Tang Edition, Image courtesy of Chinese—Japanese Library, Harvard University, **(B)** “*BaiJi*-type” Polygonati rhizoma, **(C)** “*Jiang*-type” Polygonati rhizoma, **(D)** “*Cylinder*-type” Polygonati rhizoma, **(E)** “*JiTou*-type” Polygonati rhizoma, and **(F)** atypical “*JiTou*-type” Polygonati rhizoma. Scale bar = 1 cm.

*Huangjing* was first cited in *Ming Yi Bie Lu* 名医别录 and has been used as a traditional Chinese medicine for more than 2000 years. Previous studies have shown that *P. sibiricum* and *P. cyrtonema* were mainly used as the primitive plants of *Huangjing* before the Ming Dynasty, and the use of *P. kingianum* was not recorded until the Qing Dynasty (Cheng and Wang, 2009). Traditional Chinese medicine researchers have noticed that there are differences in the morphology of rhizomes between *P. sibiricum* and *P. cyrtonema*, known as *Jiang Huangjing* (the morphology of rhizomes is similar to the rhizome of ginger, which is called *Jiang* in Chinese) and *Jitou Huangjing* (the morphology of rhizomes is similar to that of the chicken head, which is called *Jitou* in Chinese). *Ben Cao Yuan Shi* 本草原始 (completed in 1612) was the first work in Chinese history to depict the map of medicinal parts, which described three types of Polygonati rhizoma, one of which has a similar morphology to Bletillae rhizoma (the rhizomes of *Bletilla striata*; Li et al., 2007; Figure 1). Through field investigation, it was found that the rhizomes of *P. sibiricum* had two types of morphology, one resembling a chicken head, called “*Jitou*-type” Polygonati rhizoma (JTPR) and the other atypical “*Jitou*-type” Polygonati rhizoma (AJTPR). Rhizome of *P. cyrtonema* is divided (Figure 1) into three types: “*Jiang*-type” Polygonati rhizoma (JPR), similar to ginger, “Cylinder-type” Polygonati rhizoma (CPR), which is nearly regular cylindrical, and “*Baiji*-type” Polygonati rhizoma (BJPR), consistent with the type of “shape like Bletillae rhizoma” recorded in *Ben Cao Yuan Shi*. Interestingly, the quality of BJPR was considered excellent by *Han Yao Liang Lie Jian Bie Fa* (Naotarov, 1955), showing that traditional Chinese medicine experts have focused on the differences in the quality of different types of Polygonati rhizoma. However, the scientific connotation of the superior quality of BJPR has not yet been revealed.

*Huangjing* contains various chemical components, such as polysaccharides, saponins, flavonoids, and alkaloids, and other components (Tang et al., 2019; Hu et al., 2021). *Huangjing* is used as both medicine and food, and its sweet taste and nutritional components, such as free sugars, amino acids, and nucleic acids, have also attracted much attention. However, presently, trait quality is ignored when evaluating the quality of *Huangjing*. A comprehensive evaluation of quality traits of *Huangjing* can provide a scientific basis for “assessing quality by distinguishing features” and help researchers identify excellent materials for improved breeding. Therefore, a scientific method for comprehensively evaluating quality traits of *Huangjing* is needed. In this study, five different types of *Huangjing* were collected as: two rhizome types of *P. sibiricum* (JTPR and AJTPR) and three rhizome types of *P. cyrtonema* (JPR, CPR, and BJPR; Figure 1B). A multivariate statistical analysis was used to comprehensively evaluate the quality of five rhizomes of *Huangjing*, including appearance, microstructure, medicinal quality, and nutritional quality.

The appearance and microstructure of *Huangjing* were compared and analyzed using by morphological and microscopic observations and histochemical experiments. Ultraviolet and visible spectrophotometry (UV-Vis) was used to determine the total polysaccharide. The contents of free sugar content were determined using high-performance liquid chromatography coupled with a charged aerosol detector (HPLC-CAD), including fructose, glucose, and sucrose. The contents of amino acids, nucleosides, and nucleobases were determined using ultra-high performance liquid chromatography-Orbitrap-tandem mass spectrometry (UHPLC-Orbitrap-MS/MS). Furthermore, the total saponin represented by the medicinal components of *Huangjing* were also determined by UV-Vis spectroscopy. The DPPH and ABTS methods were also used to confirm the antioxidant activities in *Huangjing*. Then, principal component analysis (PCA) and orthogonal partial least squares discrimination analysis (OPLS-DA) were used to illustrate the component difference among the five types of *Huangjing*. This research aimed to provide a reliable and useful classification for clarifying the differences in plant material of different characters of *Huangjing* and provide a reference for the breeding of improved varieties and further quality evaluation of *Huangjing*.

## Materials and Methods

### Materials

Thirty-six batches (Supplementary Table 1) of *P. sibiricum* and *P. cyrtonema* were collected from two producing regions of China, including Jinzhai and Quanjiao of Anhui province. Professor Huasheng Peng authenticated all herbal samples. After collection, fresh rhizome samples were steamed for 2 h at 100°C, dried in a drying oven at 60°C until constant weight, and then ground into a fine powder (100 mesh).

### Chemicals and Reagents

Details of standard compounds (purity ≥98%), including two monosaccharides and one disaccharide, sarsasapogenin, 24 amino acids, 11 nucleosides, four nucleobases, and other compounds, are listed in Supplementary Table 2. The chemical structures of the standard compounds are shown in Supplementary Figure 1. MS-grade acetonitrile (ACN) was obtained from Merck (Darmstadt, Germany). Ammonium acetate and acetic acid (MS grade) were acquired from Sigma Chemical Co., Ltd. (St. Louis, MO, United States). Pure water was prepared using a Direct-Pure®Adept water system (RephiLe Bioscience, Ltd., Shanghai, China). All other reagents and chemicals were of analytical grade.

### Morphological and Microscopic Observations

#### Morphological Observation

Five types of *Huangjing* were observed, and the plant materials were photographed using a digital camera (Canon EOS 70D, EF 24–70 mm f/4 l IS USM, Japan). Data comprising the diameter of the extension segment fine end and thick end, diameter of stem mark, internodal distance, height, and width of buds, annular spacing, and branch angle of rhizomes were measured and recorded.

#### Microscopic Observation

Microscopic observation was performed using a previously described method (Xie et al., 2019), and paraffin sections were prepared. The fresh samples were fixed in FAA solution (70% ethanol: acetaldehyde: acetic acid = 90:5:5, v/v/v), followed by routine dehydration with a series of ethanol and paraffin embedding. A Leica RM2265 rotary microtome (Leica Microsystems Wetzlar GmbH, Wetzlar, Hessen, Germany) was used to produce sections (15 μm thick) for permanent slide preparation and stained with safranin and fast green. The microscopic characteristics of the rhizomes were examined using an optical microscope (Motic Panthera, China). The percentage of mucilage cells was calculated by freeze sectioning (Leica CM1950, Germany) combined with an ink staining experiment. Briefly, fresh rhizomes were collected, and a section was cut without pretreatment and embedded into a freezing medium; the temperature of the condensation box was −22°C. After 30 min of condensation time, the sections were sliced after trimming with a thickness of 30 μm. Six glass slides were prepared and observed for each type, and the average value was used to calculate the percentage area of mucilage cells.

#### Histochemical Analyses

Histochemical analyses were performed on polysaccharides, saponins, and proteins. Polysaccharides were stained using periodic acid-Schiff (PAS) reaction, saponins using 5% vanillin-glacial acetic acid-perchloric acid solution (Cheng and Wang, 2013), and proteins using Coomassie brilliant blue test solution (Fazekas de St Groth et al., 1963).

### Qualitative Analysis

#### Total Polysaccharide Content

Total polysaccharide was determined using the anthrone-sulfuric acid method. About 0.25 g of the as-prepared rhizome powder was weighed accurately into a round-bottom flask with 150 ml of 80% ethyl alcohol and boiled for 60 min. After removal of insoluble matter, 150 ml of water was added and boiled again for 60 min. All solutions were collected and diluted in a 250 ml volumetric flask with water. Then, 8.0 ml of 0.2% anthrone-sulfuric acid solution was added to 1 ml of sample solution and 1 ml of water. The absorbance was measured at 620 nm, and D (+)-glucose was used as a reference. Each sample was prepared in triplicate.

#### Free Sugar Content

Two monosaccharides and one disaccharide were identified using a high-performance liquid chromatography coupled with a charged aerosol detector (HPLC-CAD). The standard stock solution was prepared by adding an appropriate amount of fructose, glucose, and sucrose standards, precision weighing, and 80% ethanol was added to dissolve the stock solutions at concentrations of 2.0890, 1.0071, and 1.0568 mg/ml of stock solution, respectively. One gram of the as-prepared rhizome powders was extracted once with 80% ethanol (30 ml) by simple sonication (500 W, 30 kHz) for 1 h, steamed, and dried. Then, an appropriate amount of 75% ACN was added and dissolved in a 50 ml volumetric flask. The sample solutions were then filtered with a 0.22 μm syringe filter, and the filtrates were collected.

Samples were analyzed on an Ultimate 3,000 Liquid Chromatograph (Corona Ultra Electromist Detector, Dionex, United States); Shodex Asahipak NH_2_P-50 4E column (4.6 mm × 250 mm, 5 μm) was used for separation. The mobile phase comprised solutions A (deionized water) and B (ACN), and the gradient elution procedure was as follows: 0–20 min, 19–25% A; 20–35 min, 25–30% A; 35–40 min, and 30–19% A. The flow rate of the mobile phase was 1.0 ml/min, and the column temperature was maintained at 25°C. The injection volume was 5 μl. CAD detector settings were as follows: data acquisition frequency 5 Hz; filter 3.6 F; atomizer temperature 35°C; air N_2_; and pressure 4.328 × 105 Pa.

#### Amino Acid, Nucleoside, and Nucleobase Content

Stock solutions of the standard compounds (approximately 1 mg/ml) were prepared by dissolving approximately 1 mg of each compound in 1.0 ml of a suitable solvent. Certain amounts of the 24 amino acids and 15 nucleosides, and nucleobases standard stock solutions were mixed and diluted with 50% ACN to obtain a series of solutions at the appropriate concentrations. These solutions were used to construct calibration curves. All standard solutions were filtered through 0.22 μm cellulose membrane filters before analysis and were stored in a refrigerator at 4°C. One gram of the as-prepared sample powder was extracted once with 60% ethanol (20 ml) by sonication (500 W, 30 kHz) for 30 min and cooled to 25°C for 30 min; solvent was again added to compensate for the weight loss. Then, the supernatant was centrifuged at 13000 rpm/min for 15 min and stored at 4°C. The mixture was diluted five times with 50% ACN solution and then centrifuged at 13000 rpm/min for 15 min. The supernatant was removed through a 0.22 μm filter membrane and was injected into an HPLC column.

Chromatographic analysis was conducted on an Ultimate HPLC system (Thermo Fisher Scientific, Waltham, MA, United States). An Acquity UPLC BEH Amide (2.1 × 100 mm, 1.7 μm) column was used for analyzing all the samples. The mobile phase consisted of solutions A (2 mm ammonium acetate and 0.1% acetic acid) and B (ACN; v/v). The gradient elution procedure was as follows: 0–3 min, 10% A; 3–9 min, 10–18% A; 9–15 min, 18–20% A; 15–16 min, 20–46% A; 16–20 min, 46% A; and 20–25 min, 46–10% A. The mobile phase was set at a 0.30 ml/min flow rate, and the injection volume was 2 μl. The column temperature was maintained at 30°C.

Mass spectrometry (MS) was performed on a Q Exactive Focus Orbitrap MS system (Thermo Fisher Scientific, United States) supplied with a heated electrosprayer for ionization. Mass spectra were acquired separately in positive ionization mode in a full mass operation with a mass range of 70.0–1,050 m/z and a spray voltage of 3.4 kV. The temperature of the capillary and source was maintained at 350°C. Pressures of 50 psi and 10 psi were set for the sheath gas (N_2_) and auxiliary gas (N_2_), respectively. Sodium trifluoroacetate was used for accurate mass calibration. Mass spectra and chromatograms were acquired and processed using Xcalibur (Thermo Fisher Scientific, Waltham, MA, United States). The chemical formula for precursor and product ions was determined using the Compound Discoverer software (version 2.0).

#### Total Saponin Content

Total saponin content was analyzed using the 5% vanillin-glacial acetic acid–perchloric acid method (Zhao et al., 2020a). About 1.0 g of the as-prepared rhizome powder was extracted with 25 ml 70% ethanol ultrasonic extract for 30 min, filtered, and spin-dried in a rotary evaporator. The pellet was dissolved using 20 ml of 1% sodium hydroxide solution, and the solution was transferred to a pear-shaped separating funnel. The extract was washed three times with water-saturated n-butyl alcohol and then twice with 15 ml of n-butyl alcohol-saturated water. Furthermore, the solution was spin-dried, and the pellet was dissolved in methanol in a 5-mL volumetric flask. The prepared sample was mixed with the developer for a color reaction, using sarsasapogenin as the reference, and the wavelength was measured at 530 nm. Each sample was prepared in triplicate.

### Antioxidant Capacity Evaluation

Antioxidant capacity was evaluated using 2,2-diphenyl-1-picrylhydrazyl (DPPH) and 3-ethyl benzothiazoline-6-sulfonic acid (ABTS) assays. An extraction method with appropriate modification of the total polysaccharide was used for sample extraction.

DPPH free radical scavenging capacity was measured using the method described by Brand-Williams (Blois, 1958) with slight modifications. During the experiment, 0.1 ml of sample extracts with different concentrations (10, 20, 30, 40, and 50 mg/ml) were mixed with DPPH ethanol solution (0.1 mmol/l, 0.1 ml), added to 96-well plates for a dark reaction for 30 min, and the absorption wavelength was measured at 517 nm using a microplate analyzer (Tecan infinite 200, Tecan, China). ABTS was performed according to a previously described method, with slight modifications (Re et al., 1999). To prepare ABTS working solution, 7 mmol/l of ABTS aqueous solution was mixed with 2.45 mmol/l potassium persulfate aqueous solution at a ratio of 1:1. The solution was left to stand in the dark for 12 h and diluted with water. The absorbance value measured at 734 nm was 0.7 ± 0.05 l/(g·cm). During the experiment, 0.1 ml of sample extracts with different concentrations (10, 20, 30, 40, and 50 mg/ml) were mixed with ABTS ethanol solution (0.1 mmol/l and 0.3 ml) and added into 96-well plates for a dark reaction for 6 min. The absorbance wavelength was set to 734 nm.

The DPPH and ABTS free radical scavenging capacities were calculated using the following formula: DPPH radical scavenging ratio (%) = [1 − (A_sample_ − A_background_)/A_blank_] × 100%, where A_sample_ is the absorbance of the sample solution and DPPH solution; A_background_ the absorbance of the sample solution and ethanol solution; A_blank_ the absorbance of water and DPPH solution; and ABTS radical scavenging ratio (%) = [1 − (A_sample_ − A_background_)/A_blank_] × 100%, where A_sample_ is the absorbance of the sample solution and ABTS solution; A_background_ the absorbance of the sample solution and water; and A_blank_ the absorbance of water and ABTS solution. IC_50_ was used to represent antioxidant activity using GraphPad Prism 8.0 software (GraphPad, San Diego, United States).

### Statistical Analysis

All data are expressed as mean ± SD. Data were statistically evaluated by one-way ANOVA analysis, least significant difference (LSD) test, and principal component analysis (PCA) scores using IBM SPSS 23.0 Statistics (SPSS, Inc., United States). Correlation analysis was performed using Origin 2021 (OriginLab, United States), and partial least squares discriminant analysis (PLS-DA) was performed using SIMCA-P 14.1 (Umetrics Inc., Sweden). Heatmap clustering analysis was performed using TBtools (Chen et al., 2020).

## Results

### Morphological Characteristics

The rhizomes of *Polygonatum* plants grow one segment each year, which is called “annual rhizomes.” There are clear differences in the appearance of the annual rhizomes of the five types of *Huangjing*. The BJPR showed two continuous bifurcating branches, and the annual rhizomes were usually ovoid in shape (Figure 1B). There are two developed latent buds, one of which is long and stout; the other is short and thin, and there is an acute angle between the two buds (57.95 ± 15.56°). JPR showed two or three bifurcating branches. The annual rhizomes are cylindrical, and their latent buds are short, small, and inconspicuous (Figure 1C). CPR with indistinct branching and annual rhizomes with latent buds were underdeveloped (Figure 1D). *P. sibiricum* showed two rhizome types. One type of annual rhizome has a thick end and changes significantly from the front end, commonly known as JTPR (Figure 1E). Another kind of annual rhizome has no significant change in thickness, which is called AJTPR (Figure 1F). Detailed data of the appearance features of the five types of *Huangjing* in Supplementary Table 3.

### Microscopic Characteristics and Histochemical Localization

**Figure 2:**
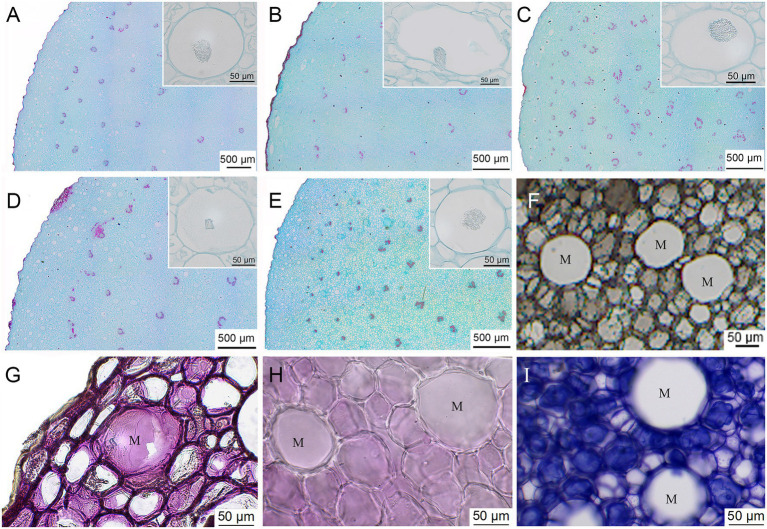
The cross-sectional microstructures of the five types of *Huangjing* **(A)** “*BaiJi*-type” Polygonati rhizoma, **(B)** “*Jiang*-type” Polygonati rhizoma, **(C)** “*Cylinder*-type” Polygonati rhizoma, **(D)** “*JiTou*-type” Polygonati rhizoma, and **(E)** atypical “*JiTou*-type” Polygonati rhizoma, ink dyeing **(F)**, histochemical localization **(G)** polysaccharides—Periodic Acid-Schiff reaction, **(H)** saponins—5% vanillin-glacial acetic acid-perchloric acid solution reaction, **(I)** proteins—Coomassie brilliant blue test reaction, and M: mucilage cell.

The cross-sectional microstructures of the five types of *Huangjing* exhibited both similarities and differences (Figures 2A–E). *P. cyrtonema* and *P. sibiricum* belong to the Liliaceae family and are typical monocotyledonous plants. The epidermis, cortex, and vascular bundle structures were found in the rhizomes of *P. cyrtonema* and *P. sibiricum*. In addition, bundles of needle-shaped calcium oxalate crystals were observed within large mucilage cells under the epidermis. The major difference among the five types of *Huangjing* was the characteristics and distribution of mucilage cells. The mucilage cells of BJPR were round, with a diameter of 30–60 μm, and were mainly distributed in the cortex near the epidermis. The number of mucilage cells in JPR was relatively small, but the individual area was large, and some of the mucilage cells appeared oval with a long diameter of up to 190 μm. The number of mucilage cells in the CPR was the lowest among the five types, and the diameter of the cells was 40–80 μm. The mucilage cells in JTPR were round, with a diameter of approximately 50 μm, and the distribution in the cortex was relatively evenly distributed. In contrast, the mucilage cells of AJTPR had varying sizes, ranging from 30 to 110 μm in diameter.

To compare the total mucilage cell area/the whole cross-sectional area ratio in the rhizomes of five kinds of *Huangjing*, frozen sections were used to section the fresh rhizomes, combined with the mucus reaction (ink dyeing), which can easily observe the mucilage cells (Figure 2F). The ratio of mucilage cell area to rhizome cross-sectional area was calculated (Supplementary Figure 2), among which the value of the BJPR was the largest (6.14 ± 1.87%), followed by the JTPR (4.61 ± 2.30%), and the value of CPR (1.17 ± 0.43%) was the lowest.

Histochemical localization techniques can visualize the distribution relationship of tissue structures and chemical components, which play a vital role in verifying the distribution of chemical components in medicinal plants (Souza et al., 2018; Ribeiro and Leitão, 2020). In this study, we used the periodic acid-schiff reaction, 5% vanillin-glacial acetic acid-perchloric acid solution, and Coomassie brilliant blue test solution to study the histochemistry of polysaccharides, saponins, and proteins in the rhizomes of *Huangjing*, respectively. The results (Figures 2G–I) showed that polysaccharides in the rhizomes of *Huangjing* were mainly distributed in mucilage cells, and saponins and proteins were mainly distributed in parenchymal cells. There were no saponins or proteins in the mucilage cells. This result is consistent with previous studies (Cheng and Wang, 2013). Thus, it is expected that BJPR may have higher concentrations of polysaccharides (depending on the mucilage cells).

### Nutritional and Medicinal Component Analysis

#### Sugars

**Figure 3:**
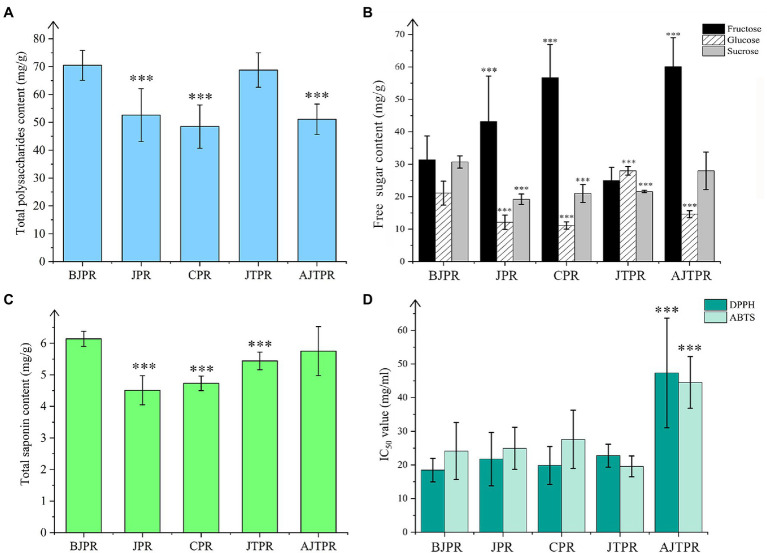
Total polysaccharide content of the five different types of *Huangjing* **(A)**; Three sugars (fructose, glucose, and sucrose) content of the five different types of *Huangjing* **(B)**; total saponin content of the five different types of *Huangjing* **(C)**; DPPH and ABTS of antioxidant ability of the five different types of *Huangjing* **(D)**. BJPR, “*BaiJi*-type” Polygonati rhizoma; JPR, “*Jiang*-type” Polygonati rhizoma; CPR, “*Cylinder*-type” Polygonati rhizoma; JTPR, “*JiTou*-type” Polygonati rhizoma; and AJTPR, atypical “*JiTou*-type” Polygonati rhizoma (^***^compared with BJPR, *p* < 0.05).

There are abundant polysaccharides in *Huangjing*, which is recognized as the evaluation index quality (Jiang et al., 2017; Li et al., 2018a). The total polysaccharide content of the five different types of *Huangjing* is shown in Figure 3A.

Among the five types, the polysaccharides content of the BJPR (70.5 ± 5.37 mg/g) and JTRP (68.8 ± 6.20 mg/g) was relatively higher. In addition, the polysaccharide content was not significantly different between BJPR and JTRP. Furthermore, the polysaccharide content in the CPR was the lowest (48.5 ± 7.75 mg/g). These results indicate that the different types of *Huangjing* samples are not uniform in polysaccharides. Interestingly, BJPR contains abundant mucilage cells. This further confirms that polysaccharides in the rhizomes of Polygonatum plants are distributed in mucilage cells. Furthermore, the total polysaccharide content showed a positive relationship with the total area of the mucilage cells.

Sweetness can improve flavor when *Huangjing* is used as a food product. The sensation of “sweetness” is determined by the presence of free sugars. Fructose, glucose, and sucrose have been reported to contribute to the sweetness of foods, and fructose is considered the sweetest sugar (Georgelis et al., 2018). Therefore, these three free sugars were often used as one of the flavor indexes for evaluating fruit (Filip et al., 2016). The HPLC-CAD method was successfully applied to quantify fructose, glucose, and sucrose in five types of *Huangjing* samples (Supplementary Figure 3). The method was validated by determining linearity, precision, stability, and accuracy. The results are summarized in Supplementary Table 4. The calibration curves exhibited excellent linear regression (R^2^ > 0.9980). The precision, repeatability, and stability variation (RSD) were all less than 2.98%. The recoveries were between 94.0 and 103.9%, with an RSD of less than 3.35%.

The contents of fructose, glucose, and sucrose were different in the five types of *Huangjing*, which may be caused by differences in the biosynthesis process, which affects their accumulation (Zhang et al., 2018a). The different types are listed in the order of most to least: fructose content: AJTPR > CPR > JPR > BJPR > JTPR, glucose content: JTPR > BJPR > AJTPR > JPR > CPR, and sucrose content: BJPR > AJTPR > JTPR > CPR > JPR. The total content of the three free sugars was the highest in the AJTPR samples; thus, we speculated that the AJTPR had a relatively sweet taste among the five types, but the specific taste of the five types of *Huangjing* still needs further flavor research.

#### Amino Acids, Nucleosides, and Nucleobases

Amino acids, nucleosides, and nucleobases are primary metabolites that play an important regulatory role in human health. The importance of amino acids, nucleosides, and nucleobases is widely recognized in food science and nutrition (Hoffer, 2016; Kuś and Rola, 2021). This study established an ultrahigh-performance liquid chromatography-Orbitrap-tandem mass spectrometry (UHPLC-Orbitrap-MS/MS) method under the positive MS ion mode to analyze 39 compounds, including amino acids, nucleosides, and nucleobases. The method was validated to determine the linearity, limits of detection (LOD) and quantitation (LOQ), precision, stability, and accuracy, and the results are summarized in Supplementary Table 6. Typical chromatograms of the standards are shown in Supplementary Figure 4. These results showed that this method could determine amino acids, nucleosides, and nucleobases in *Huangjing.*

Among the 24 free amino acids, nine essential amino acids (phenylalanine, methionine, lysine, leucine, isoleucine, tryptophan, threonine, valine, and histidine) are either not synthesized by the human body or synthesized at a rate far from being adapted to the needs of the organism and must be supplied through food (Church et al., 2020). In addition, amino acids are hydrophilic compounds that play a key role in forming umami, sweet, and bitterness in foods (Wang et al., 2021a). The results (Table 1) showed that umami amino acids (glutamate and aspartic acid) accounted for 46.2% of the total content of 24 amino acids, sweet amino acids (threonine, serine, glycine, alanine, and methionine) for 6.4%, and bitter amino acids (valine, isoleucine, leucine, tyrosine, phenylalanine, and lysine) for 3.7% in all *Huangjing* samples. The total content of 24 amino acids exhibited different patterns and reached the highest in the JTPR (64.3 ± 0.43 mg/g). Among the 24 amino acids from *Huangjing* samples, aspartic acid and citrulline are the most abundant and play crucial roles in nutrient metabolism pathways such as the ornithine cycle (Sivashanmugam et al., 2017). Interestingly, the content of aspartic acid was highest in JTPR (53.4%), and citrulline was the most abundant in BJPR (46.1%).

**Table 1:** Contents of 24 amino acids and 15 nucleosides and nucleobases (μg/g) of the five different types of *Huangjing.*

	BJPR	JPR	CPR	JTPR	AJTPR	Mean
L-Cysteine	45.43 ± 2.41b	34.37 ± 3.21c	33.64 ± 2.71c	41.55 ± 1.56bc	66.78 ± 14.78a	42.69 ± 13.25
L-Phenylalanine	198.35 ± 14.76a	143.7 ± 12.21b	71.85 ± 28.01c	221.63 ± 19.83a	213.43 ± 71.55a	165.44 ± 61.34
L-Alanine	36.93 ± 4.39a	24.39 ± 15.43a	31.63 ± 14.81a	45.21 ± 44.58a	24.02 ± 4.38a	31.1 ± 21.44
Citrulline	22251.03 ± 4133.4a	15148.6 ± 2003.43b	5581.18 ± 3359.44c	18649.09 ± 1460.93ab	6399.16 ± 3277.71c	13862.94 ± 6709.49
L-Glutamine	1312.17 ± 625.21a	69.44 ± 57.42b	24.87 ± 16.19b	157.56 ± 26.16b	378.53 ± 232.06b	335.33 ± 524.2
L-Glutamic acid	1149.71 ± 201.78a	502.34 ± 180.04c	218.85 ± 62.17d	876.18 ± 124.03b	398.69 ± 97.11 cd	608.02 ± 346.46
L-Methionine	101.24 ± 22.51ab	80.46 ± 24.61bc	44.26 ± 50.26c	124.96 ± 8.18ab	140.52 ± 81.27a	95.32 ± 51.05
L-Arginine	375.67 ± 36.53a	279.47 ± 33.16b	112.79 ± 50.95c	343.99 ± 18.6a	130.12 ± 54.89c	253.58 ± 107.63
Lysine	1223.73 ± 210.66a	619.97 ± 108.23b	139.47 ± 73.58c	1254.32 ± 152.26a	261.27 ± 84.88c	686.45 ± 451.33
L-Tyrosine	70.65 ± 13.26a	24.66 ± 4.05b	21.6 ± 6.54b	60.1 ± 11.47a	21 ± 4.22b	37.11 ± 21.91
L-Leucine & L-Isoleucine	279.21 ± 25.59b	187.93 ± 24.52c	96.24 ± 43.22d	398.36 ± 31.97a	234.14 ± 119.44bc	230.63 ± 108.09
L-Ornithine	102.36 ± 20.18a	66.19 ± 15.33b	39.43 ± 8.21c	68.58 ± 4.45b	33.46 ± 2.51c	62.7 ± 25.78
Taurine	0.05 ± 0.05a	–	–	0.01 ± 0.01b	–	0.01 ± 0.03
L-Proline	1725.74 ± 118.91a	748.62 ± 209.51bc	652.86 ± 338.13c	964.09 ± 56.78b	367.69 ± 134.69d	867.94 ± 467.59
L-Hydroxyproline	37.43 ± 2.47c	63.5 ± 19.93b	28.77 ± 7.98c	121.1 ± 16.33a	42.55 ± 6.96c	59.47 ± 33.62
GABA	401.94 ± 26.04a	177.8 ± 19.25b	81.03 ± 36.17c	371.17 ± 85.65a	227.73 ± 45.14b	239.58 ± 121.94
L-Tryptophan	62.97 ± 8.05b	42.23 ± 9.57b	49.11 ± 31.93b	192.56 ± 15.04a	162.16 ± 61.76a	91.88 ± 68.17
L-Serine	348.94 ± 29.43a	297.22 ± 67.33a	136.06 ± 13.11c	319.58 ± 50.06a	217.85 ± 20.62b	269.48 ± 85.3
L-Threonine	1606.33 ± 387.23b	1691.33 ± 486.47b	2525.28 ± 712.23a	3117.94 ± 507.72a	1482.78 ± 187.07b	2019.16 ± 759.18
L-Aspartic acid	13760.68 ± 2601.42bc	9369.74 ± 2806.93c	10561.96 ± 4465.84c	34592.54 ± 6864.92a	17996.39 ± 3163.24b	15941.84 ± 9767.03
Asparagine	2590.56 ± 195.99a	530.87 ± 163.39d	175.47 ± 110.55e	1855.13 ± 220.2b	820.13 ± 159.08c	1083.84 ± 880.64
L-Valine	401.29 ± 13.83a	205.34 ± 24.59b	147.59 ± 54.28b	418.96 ± 45.71a	195.66 ± 67.71b	262.36 ± 115.06
Histidine	198.13 ± 51.34a	111.23 ± 9.39b	39.36 ± 12.04c	126.44 ± 11.51b	110.12 ± 10.65b	116.09 ± 51.53
2′-Deoxyadenosine	0.58 ± 0.11a	0.11 ± 0.06b	0.02 ± 0.03b	0.49 ± 0.44a	0.02 ± 0.03b	0.22 ± 0.29
dAMP	1.12 ± 1.19	–	–	–	–	0.19 ± 0.62
2′-Deoxyinosine	0.31 ± 0.04a	0.1 ± 0.02b	0.06 ± 0.02b	0.27 ± 0.2a	0.06 ± 0.01b	0.15 ± 0.13
Cytidine	2.5 ± 1.2b	2.99 ± 2.27b	1.19 ± 0.35b	12 ± 8.52a	1.71 ± 0.62b	3.9 ± 5.12
Cytidine −5- monophosphate	13.33 ± 1.45b	10.92 ± 1.54c	7.9 ± 1.12d	16.43 ± 1.47a	9.96 ± 1.1c	11.57 ± 3.03
Cytosine	0.92 ± 0.15a	0.75 ± 0.1ab	0.61 ± 0.23b	0.86 ± 0.17a	0.9 ± 0.22a	0.8 ± 0.19
cGMP	3.9 ± 1.34bc	6.3 ± 3.24ab	1.43 ± 1.04c	10.01 ± 6.44a	3.29 ± 0.96bc	5.2 ± 4.19
cAMP	12.48 ± 4.67b	17.88 ± 7.63b	4.55 ± 4.29b	36.87 ± 22.36a	9.06 ± 1.63b	16.45 ± 14.3
Inosine	29.93 ± 5.34ab	27.05 ± 7.25abc	16.3 ± 2.96c	36.29 ± 17.32a	23.86 ± 1.24bc	26.75 ± 10.12
Inosine-5′-monophosphoric acid	261.01 ± 40.65b	231.29 ± 51.22b	122.91 ± 42.53c	326.01 ± 30.11a	295.26 ± 57.89ab	244.63 ± 78.42
Guanosine	10.06 ± 3.31ab	7.16 ± 4.29abc	2.39 ± 1.14c	10.49 ± 7.69a	4.69 ± 1.08bc	6.99 ± 4.94
Guanine	2.17 ± 0.22a	0.82 ± 0.32b	0.4 ± 0.22b	1.88 ± 1.2a	0.49 ± 0.15b	1.1 ± 0.85
Adenosine	18.98 ± 4.29ab	16.88 ± 5.1abc	8.85 ± 1.8c	24.28 ± 13.93a	15.01 ± 0.95bc	16.81 ± 7.8
Adenine	2.95 ± 0.31a	1.75 ± 0.62b	0.94 ± 0.37c	1.78 ± 0.59b	1.74 ± 0.34b	1.82 ± 0.76
Thymine	1.21 ± 0.22a	0.22 ± 0.25bc	0.11 ± 0.17bc	0.46 ± 0.54b	-	0.37 ± 0.49

*BJPR, “BaiJi-type” Polygonati rhizoma; JPR, “Jiang-type” Polygonati rhizoma; CPR, “Cylinder-type” Polygonati rhizoma; JTPR, “JiTou-type” Polygonati rhizoma; and AJTPR, atypical “JiTou-type” Polygonati rhizoma.*

Studies have reported that nucleosides and nucleotides can bind to taste receptors to improve taste. For example, inosine 5′-monophosphate (IMP) and guanosine 5′-monophosphate (GMP) can cause umami taste and adenosine 5′-monophosphate (AMP) enhances sweetness (Wang et al., 2021c). Coincidentally, with respect to the total contents of 15 nucleosides and nucleotides, the highest concentration was also in the JTPR (478 ± 4.93 μg/g). This provides new insights for further research on the flavor of *Polygonatum* plants.

#### Total Saponin

*Polygonatum* plants have several steroidal saponins and fewer triterpenoid saponins, and total saponin have attracted more attention (Zhao et al., 2018a). The total saponin contents of the samples from different types of *Huangjing* are shown in Figure 3C. The total content of saponins in the BJPR (6.14 ± 0.237 mg/g) and AJTPR(5.75 ± 0.777 mg/g) was significantly higher than that in the other types. Therefore, it can be intuitively seen that it is impractical to directly compare the quality of different types of *Huangjing* using an index component.

### Antioxidant Capacity Analysis

*Huangjing* has an antioxidant capacity (Zhang et al., 2019); however, there are few reports on the antioxidation of different types. Based on the results, the contents of primary and secondary metabolites of different types of *Huangjing* were different, so we speculated that their antioxidant capacities were also different. DPPH and ABTS radicals have been widely used to analyze the preliminary free radical scavenging capacity of plant extracts or antioxidant compounds (Xie et al., 2014). To evaluate the antioxidant capacity of *Huangjing* more comprehensively, we used rapid DPPH and ABTS radical scavenging assays. DPPH evaluated the antioxidant capacity of fat-soluble components, and ABTS evaluated the antioxidant capacity of water-soluble and fat-soluble components. The DPPH and ABTS radical scavenging activities of the five types of *Huangjing* were expressed as IC_50_ values and are shown in Figure 3D. A higher IC_50_ value indicates weaker antioxidant capacity. In contrast, the lower the IC_50_ value, the stronger the antioxidant capacity. The values showed that the antioxidant activity gradually increased with increased sample concentration and had a strong scavenging effect on DPPH and ABTS. The scavenging rates were 88.8 and 90.1%, respectively. Combined with the results of the DPPH and ABTS assays, the IC_50_ values of AJTPR were significantly higher than those of the other four types, indicating that the antioxidant capacity of AJTPR was significantly lower than that of the other four types. BJPR showed the strongest inhibition ability of DPPH, and CPR showed the strongest ABTS radical scavenging ability. Although ABTS and DPPH antioxidant experiments have some limitations, they can also provide a reference for further evaluation of the quality of *Huangjing*.

### Heatmap Cluster Analysis

**Figure 4:**
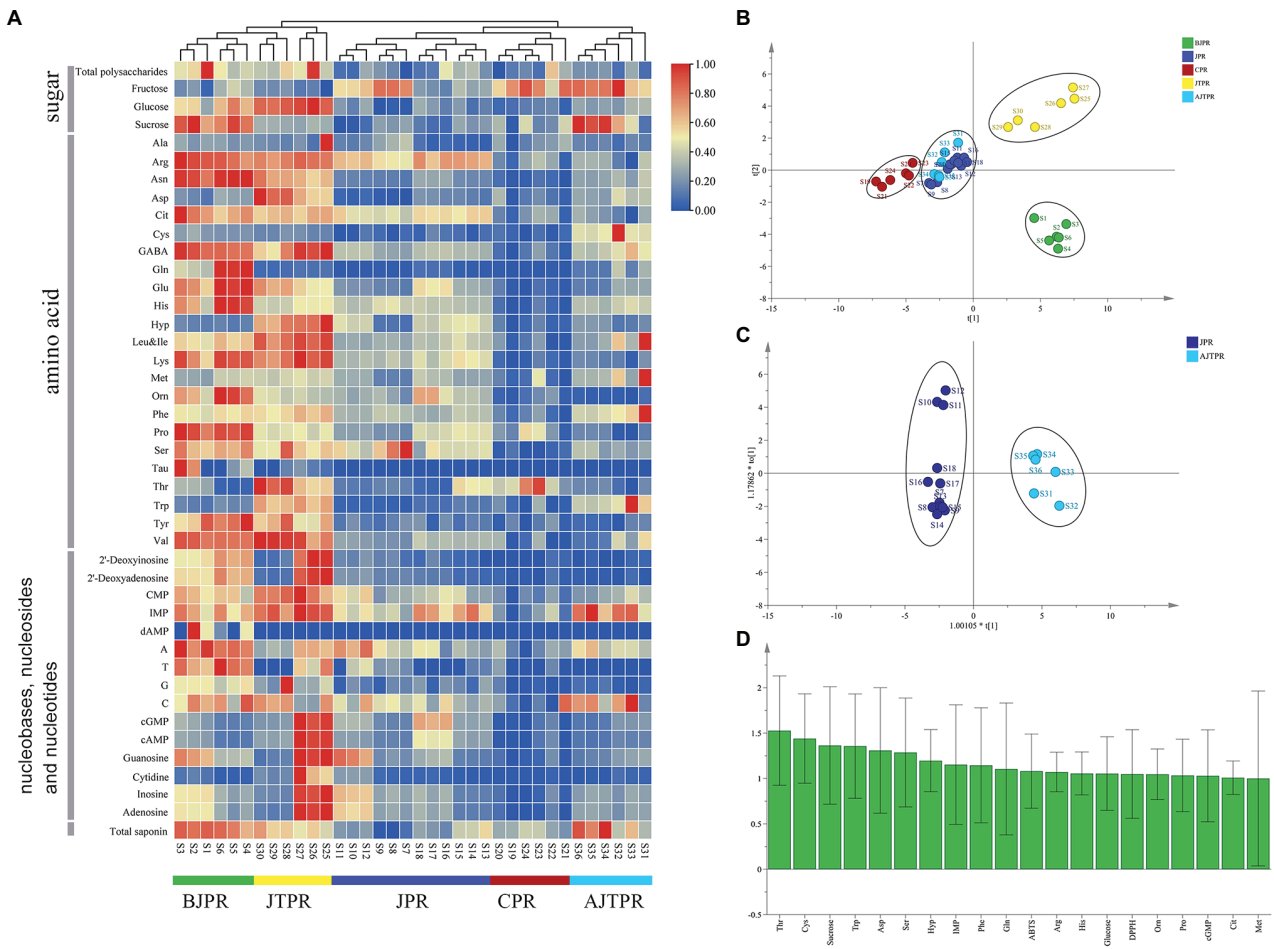
Heatmap cluster analysis and multivariate statistical analysis **(A)** Hierarchical clustering analysis heat maps, **(B)** PLS-DA score plot, **(C)** OPLS-DA score plot, and **(D)** the VIP plot. BJPR, “*BaiJi*-type” Polygonati rhizoma; JPR, “*Jiang*-type” Polygonati rhizoma; CPR, “*Cylinder*-type” Polygonati rhizoma; JTPR, “*JiTou*-type” Polygonati rhizoma; and AJTPR, atypical “*JiTou*-type” Polygonati rhizoma.

A heat map can directly show intragroup and intergroup similarities and differences, and cluster analysis is a clustering tool to evaluate the difference through an algorithm to produce a dendrogram (Yang et al., 2021). Thus, heatmap cluster analysis with complete linkage after column scaling with normalization was used in this study to evaluate the similarities and differences of different types of *Huangjing*. The results (Figure 4A) showed that the five types of *Huangjing* were classified into two clusters and exhibited significant spatial aggregation. The colors represent the level of accumulation for each metabolite, from low (blue) to high (red). Heat maps show that more than 60% of the compounds were higher in the BJPR and JTPR. The samples of BJPR and JTPR were grouped into Cluster I. The other samples, including those from JPR, CPR, and AJTPR, were grouped into Cluster II and exhibited a lower content of total polysaccharide, total saponin, glucose, sucrose, amino acids, nucleosides, and nucleobases. Based on these results, the comprehensive quality of the BJPR and JTPR is better. Nevertheless, it is necessary to further evaluate the quality characteristics of different *Huangjing* sample types using chemometrics.

### Multivariate Statistical Analysis

The analysis of total polysaccharide, total saponin, free sugars, amino acids, nucleosides, and nucleobases contents showed that the differences in various components were found in the five types of *Huangjing*. Therefore, PCA and OPLS-DA analyses were used to determine which components had the greatest effect on the differences and could be used as differential compounds to distinguish them. However, PCA is an unsupervised multivariate statistical method of pattern recognition, which cannot assign the class membership of unknown test samples; therefore, it has limited practical use. In contrast, OPLS-DA is a multivariate dimensionality reduction tool and a supervised pattern recognition method that is gaining popularity in metabolomics and other integrative omics analyses (Ruiz-Perez et al., 2020).

After the chemical component data were standardized, the principal component score was calculated, which were the major differential compounds with variable importance in projection (VIP) values greater than 1. Through the principal component score, four principal components were extracted with eigenvalues greater than 1. The characteristic values of the four principal components were 7.253, 4.010, 3.665, and 1.563, respectively. The cumulative contribution rate of the four principal components was 82.5%. Four principal components were selected as the main evaluation factors, which comprehensively evaluated the quality of *Huangjing* (Supplementary Table 8). From the principal component score, the quality of the BJPR is better than that of the other types of *Huangjing*, followed by the JTPR.

However, because PCA cannot assign the class membership of unknown test samples, critical to validating statistical models, OPLS-DA extends a regression of PCA and uses class information to maximize the separation between groups of observations, thereby allowing better classification and prediction capacity. The OPLS-DA quality of the models was described using the statistical parameters R^2^Y and Q^2^. Generally, the value of R^2^Y varies from 0 to 1, where 1 indicates a model with a perfect fit. Q^2^ values of >0.5 indicate excellent predictive abilities. The PLS-DA model established in the first step (Figure 4B), the model’s statistical parameters R^2^Y (0.936) and Q^2^ (0.907) were significant, and the five types of *Huangjing* were divided into four groups: BJPR, CPR, and JTPR were clearly separated, but the other two types (JPR and AJTPR) were not. Next, the OPLS-DA model was established (Figure 4C), the OPLS-DA is suitable for modeling between two groups (Ma et al., 2020), and the score plot showed a clear separation between the two types of samples (JPR and AJTPR). In the established statistical model, the R^2^Y (0.978) and Q^2^ (0.947) values were greater than 0.9, indicating the excellent quality of the OPLS-DA model. The VIP is an index used to understand the importance of spectral variables in defining the latent variable subspace and was used to evaluate the importance of the variables in the projection of the PLS-DA model. A VIP value >1 was considered to carry the most relevant information for class discrimination (Wang et al., 2021b). The VIP value (Figure 4D) illustrated that threonine (Thr), cysteine (Cys), sucrose, tryptophan (Trp), aspartic acid (Asp), serine (Ser), hydroxyproline (Hyp), inosine 5′-monophosphate (IMP), phenylalanine (Phe), glutamine (Gln), ABTS, arginine (Arg), Histidine (His), glucose, DPPH, Ornithine (Orn), Proline (Pro), Cyclic guanosinc monophosphate (cGMP), Citrulline (Cit), and Methionine (Met) were the main markers discriminating different types of *Huangjing* and contributing to the differences among the samples from different types (VIP > 1).

## Discussion

Rhizoma polygonati (*Huangjing*) has a long history of medicinal use in China. Ancient Chinese medicine experts had long noticed the diversity of rhizomes of *Huangjing*. *Ben Cao Yuan Shi* of the Ming Dynasty painted the rhizome of *Huangjing*, including BJPR. Japanese scholars regarded as BJPR had excellent quality. According to the 2020 Edition of Chinese Pharmacopoeia, *Polygonatum sibiricum* Red., *P. kingianum* Coll. et Hemsl., and *P. cyrtonema* Hua are the authentic sources of Rhizoma polygonati (The State Pharmacopoeia Committee of China, 2020). On the basis of the textual research records of materia medica, *P. kingianum* was recorded for the first time on *An Illustrated Book on Plants* (AD1848), while *P. cyrtonema* and *P. sibiricum* had been widely used in the Tang and Song dynasties (Cheng and Wang, 2009; Xu et al., 2022). Based on the records in *Ben Cao Yuan Shi* and *Han Yao Liang Lie Jian Bie Fa*, the rhizomes of *Huangjing* had different types. Recent years, many researchers had studied the chemical constituents of *Huangjing* (Fan et al., 2020; Zhao et al., 2020b; Hu et al., 2021). Indeed, some scholars have paid attention to the deeper material activity of *Huangjing*, it contains a significant number of nanoparticles that can be used as herbzymes (Benassi et al., 2021). However, the relationship between rhizome type and its quality and special metabolites was neglected. Anhui Province is one of the main producing areas of *Huangjing*. This study investigated and collected a variety of rhizomes of *P. cyrtonema* and *P. sibiricum* for comparative research of special metabolites, including the BJPR mentioned in these herbal books.

Since ancient times, *Huangjing* had been suitable for both medicine and food. Its rhizome is rich in chemical ingredients, including nutritional ingredients, such as sugars and amino acids, and medicinal ingredients such as saponins. At present, polysaccharides of *Huangjing* have attracted much attention of researchers (Jin et al., 2018; Fan et al., 2020; Hu et al., 2021). It had been reported that monosaccharides cannot be detected by a UV detector without derivatization due to the particularity of functional group structure. Fan et al. studied the monosaccharides of mannose, galacturonic acid, glucose, ribose, galactose, fucose, glucose acid, rhamnose, and arabinose in *Huangjing* by precolumn derivatization integrated with HPLC-MS/MS. However, precolumn derivatization has the limitation of incomplete derivatization reaction. Jin et al. determined fructose, glucose, galactose, sucrose, and 1-kestose in *Huangjing* by HPLC-QTOF-MS/MS, which can accurately identify the structure of compounds with high sensitivity, but it also has the limitations of complex operation and needs for professional guidance. To avoid the measurement error caused by derivatization for quantitative determination these compounds using UV detector, the detector of CAD has been used as a reliable and highly sensitive detector to low molecular weight non-volatile compounds (Jing et al., 2014). That is why this study chose this detection method, HPLC combined with CAD detector, a simple, easy to operate, high sensitivity, and no need for pre column derivatization and UV detector. In this study, three sugars (fructose, glucose, and sucrose) in *Huangjing* were determined. In addition, the content of total polysaccharide was also determined by UV-vis spectrophotometry. The results showed that the contents of total polysaccharide and three sugars in rhizomes of different types of *Huangjing* were different (Figures 3A,B). The content of fructose was the highest in AJTPR, the content of glucose was the highest in JTPR, and the content of sucrose and total polysaccharide was the highest in BJPR. Furthermore, saponin components from *Polygonatum* plants have important pharmacological effects, such as anti-inflammatory, anti-tumor, hypoglycemic, and hepatoprotective effects and modulation of the intestinal flora (Zhang et al., 2018b; Ma et al., 2019; Luo et al., 2020; Chai et al., 2021; Li et al., 2021b; Wang et al., 2021b). This study analyzed the content of saponins and found that the content of saponins was less in *Huangjing*. Among the five types of *Huangjing*, the content of saponins in BJPR was the highest (Figure 3C). It is speculated that BJPR might have better medicinal value.

Amino acids, nucleosides and nucleobases are indispensable to biological cells to sustain life activities and have good physiological activity (Pu et al., 2020). The determination of amino acids has been paid attention to in traditional Chinese medicine such as Goji berries, Angelicae sinensis radix, Semen sojae praeparatum, and Safflower so far (Chai et al., 2017; Qu et al., 2019; Pu et al., 2020; Lu et al., 2021). Therefore, it is meaningful to clarify the composition of nucleosides and amino acids in *Huangjing* for understanding the edible value of it. Some scholars had quantified seven amino acids in *P. verticillatum* (Sharma et al., 2021). However, there is no research on determining the nucleosides, nucleobases, and amino acids in *P. cyrtonema* and *P. sibiricum* recently. Comparing with various profiling techniques, Orbitrap is one of widely used mass spectrometry technique in metabolomics research, because it has the ability of a non-targeted search and fragmentation (Li et al., 2021a). In this study, UHPLC-Orbitrap-MS/MS was established for comparatively analyzing five types of *Huangjing* on amino acids, nucleosides, and nucleobases. A method for simultaneous determination of 39 amino acids, nucleosides, and nucleobases was established for the first time. The results (Figure 4A) showed that the contents of amino acids, nucleosides, and nucleobases were different in five types of *Huangjing.*

*Huangjing* is often used as food, and its antioxidant capacity has also attracted much attention. Studies had confirmed that polysaccharide of *Huangjing* has antioxidant capacity (Li et al., 2018b; Zhang et al., 2019). DPPH radical scavenging activity and ABTS radical scavenging activity are widely used for evaluated the activities of antioxidants, which are classified to the electron transfer mechanism. The antioxidant activities of *Huangjing* were evaluated by DPPH radical scavenging activity assay and ABTS radical scavenging activity assay *in vitro*. The results (Figure 3D) showed that the antioxidant capacity of different types was various, and the antioxidant capacity of AJTPR was the weakest.

Above all, the nutritional value of *Huangjing* is as important as its medicinal value. The five rhizome types of *Huangjing* were compared in sugar, saponins, amino acids, and antioxidant capacity. It was found that BJPR was more prominent in total sugar, total saponins, and most amino acids content. The other rhizome types also have their advantages, such as glucose content, total content of 24 amino acids, and total content of 15 nucleosides, and nucleobases were the highest in JTPR, ABTS radical scavenging activity was the strongest in JTPR. Fructose content was the highest in AJTPR. These also stated different types of rhizomes of *Huangjing* on chemical component content and antioxidant capacity had different levels, and the rhizome quality of different types also had its own advantages. Therefore, it is necessary to distinguish the rhizome types of *Huangjing* in medicine and food. This study provides reference basis for further quality breeding and quality improvement of *Huangjing*.

## Conclusion

This study systematically investigated the metabolites using multidimensional analysis technology and comprehensively evaluated the quality of different types of *Huangjing*, including BJPR, JPR, CPR, JTPR, and AJTPR. Based on the microstructure, the histochemical localization of polysaccharides, saponins, and proteins in *Huangjing* was conducted. The contents of total polysaccharide and saponin were determined by UV-visible spectroscopy. We found that these contents were related to the mucilage cell area/the whole cross-sectional area ratio. A reliable, simple, and sensitive method capable of quantifying 24 amino acids and 15 nucleosides and nucleobases was established using the UHPLC-Orbitrap-MS/MS method. Meanwhile, an HPLC-CAD method was established to determine the three types of sugars. The antioxidant capacity was investigated using DPPH and ABTS assays. The multidimensional evaluation results indicated that the comprehensive quality of BJPR and JTPR is better, regardless of nutritional or medicinal values. Certainly, other rhizome types also have advantages. AJTPR and CPR were categorized as the high sweet taste rhizomes based on the fructose content. Nevertheless, the perceived flavor of *Huangjing* resulting from the combination of various taste and mouthfeel sensations and the flavor evaluation indexes need to be further studied. But these results provide new insights for further research on the flavor of *Huangjing*. In general, this study proposed methods to accurately determine the contents of total polysaccharide, total saponin, three sugars, and 39 metabolites in *Huangjing*. The quantitative and comparative results revealed the chemical characteristics and differences of different types of *Huangjing*, providing a basis for germplasm utilization and related food development in *Huangjing*.

## Data Availability Statement

The original contributions presented in the study are included in the article/Supplementary Material, further inquiries can be directed to the corresponding authors.

## Author Contributions

YH and MYi performed the experiments and writing—original draft. The experiments were carried out under the supervision of YB, SC, and LZ. YH, MYa, and XZ validated and analyzed the data. ZY investigated and collected the samples. JL and LL provided resources. LH and HP conducted writing—review and editing. All authors contributed to the article and approved the submitted version.

## Funding

This study is financially supported by the National Key Research and Development Program of China (grant no. 2017YFC1701601), Scientific and technological innovation project of China Academy of Chinese Medical Sciences (CI2021A04001), CAMS Innovation Fund for Medical Sciences (2019-I2M-5-065), and Innovation Team and Talents Cultivation Program of National Administration of Traditional Chinese Medicine (No: ZYYCXTD-D-202005).

## Conflict of Interest

LL was employed by Jinzhai Senfeng Agricultural Technology Development Co., Ltd.

The remaining authors declare that the research was conducted in the absence of any commercial or financial relationships that could be construed as a potential conflict of interest.

## Publisher’s Note

All claims expressed in this article are solely those of the authors and do not necessarily represent those of their affiliated organizations, or those of the publisher, the editors and the reviewers. Any product that may be evaluated in this article, or claim that may be made by its manufacturer, is not guaranteed or endorsed by the publisher.

## Supplementary Material

The Supplementary Material for this article can be found online at https://www.frontiersin.org/articles/10.3389/fpls.2022.891775/full#supplementary-material

## Abbreviation

ABTS: 3-Ethyl Benzothiazoline-6-Sulfonic Acid
ACN: Acetonitrile
AJTPR: Atypical “Jitou-type” Polygonati rhizoma
AMP: Adenosine 5’-Monophosphate
Arg: Arginine
Asp: Aspartic acid
BJPR: “Baiji-type” Polygonati rhizoma
cGMP: Cyclic Guanosinc Monophosphate
Cit: Citrulline
CPR: “Cylinder-type” Polygonati rhizoma
Cys: Cysteine
DPPH: 2,2-Diphenyl-1-Picrylhydrazyl
Gln: Glutamine
GMP: Guanosine 5’-Monophosphate
His: Histidine
HPLC-CAD: High-Performance Liquid Chromatography Coupled with a Charged Aerosol Detector
Hyp: Hydroxyproline
IMP: Inosine 5’-Monophosphate
JPR: “Jiang-type” Polygonati rhizoma
JTPR: “Jitou-type” Polygonati rhizoma
LSD: Least Significant Difference
Met: Methionine
OPLS-DA: Orthogonal Partial Least Squares Discrimination Analysis
Orn: Ornithine
PAS: Periodic Acid-Schiff Reaction
PCA: Principal Component Analysis
Phe: Phenylalanine
Pro: Proline
Ser: Serine
Thr: Threonine
Trp: Tryptophan
UHPLC-Orbitrap-MS/MS: Ultra-High High-Performance Liquid Chromatography-Orbitrap-tandem Mass Spectrometry
UV–Vis: Ultraviolet and Visible Spectrophotometry
VIP: Variable Importance in Projection.
