# Supplementary material for: An Evaluation of Traits, Nutritional, and Medicinal Component Quality of Polygonatum cyrtonema Hua and P. sibiricum Red
Source: Front Plant Sci. 2022 Apr 18;13:891775. doi: 10.3389/fpls.2022.891775 (PMC9062581; doi:10.3389/fpls.2022.891775)
Supplement: Supplementary file 1 [file Data_Sheet_1.docx]

***Supplementary Material***

**Figures and Tables**

**Supplementary Figure 1.** Chemical structures of the standard compounds.

**Supplementary Figure 2.** The ratio of mucilage cell area to rhizome cross-sectional area (%).

**Supplementary Figure 3.** High-pertypeance liquid chromatography coupled with charged aerosol detector (HPLC-CAD) chromatogram of mix standards (a) and samples (b) for 3 sugars of *Huangjing*.

**Supplementary Figure 4.** Ultra-high pertypeance liquid chromatography-Orbitrap-tandem mass spectrometry (UHPLC-Orbitrap-MS/MS) chromatogram of 24 amino acids, 15 nucleosides and nucleobases.

**Supplementary Table** **1.** The collecting places of *P. cyrtonema* and *P. sibiricum*.

**Supplementary Table** **2.** Detailed information of standards.

**Supplementary Table** **3.** Detailed data of the appearance features of the five types of *Huangjing.*

**Supplementary Table** **4.** Regression curves, precision, repeatability, stability and recovery of 3 sugars.

**Supplementary Table** **5.** The ion mode and parameters for MRM of 24 amino acids, 15 nucleosides and nucleobases analytes.

**Supplementary Table** **6.** Regression curves, Linear range, LOD, LOQ of 24 amino acids, 15 nucleosides and nucleobases analytes.

**Supplementary Table** **7.** Precision, repeatability, stability and recovery of 24 amino acids, 15 nucleosides and nucleobases analytes.

**Supplementary Table** **8.** The comprehensive score and ranking of *Huangjing* from different types by PCA.


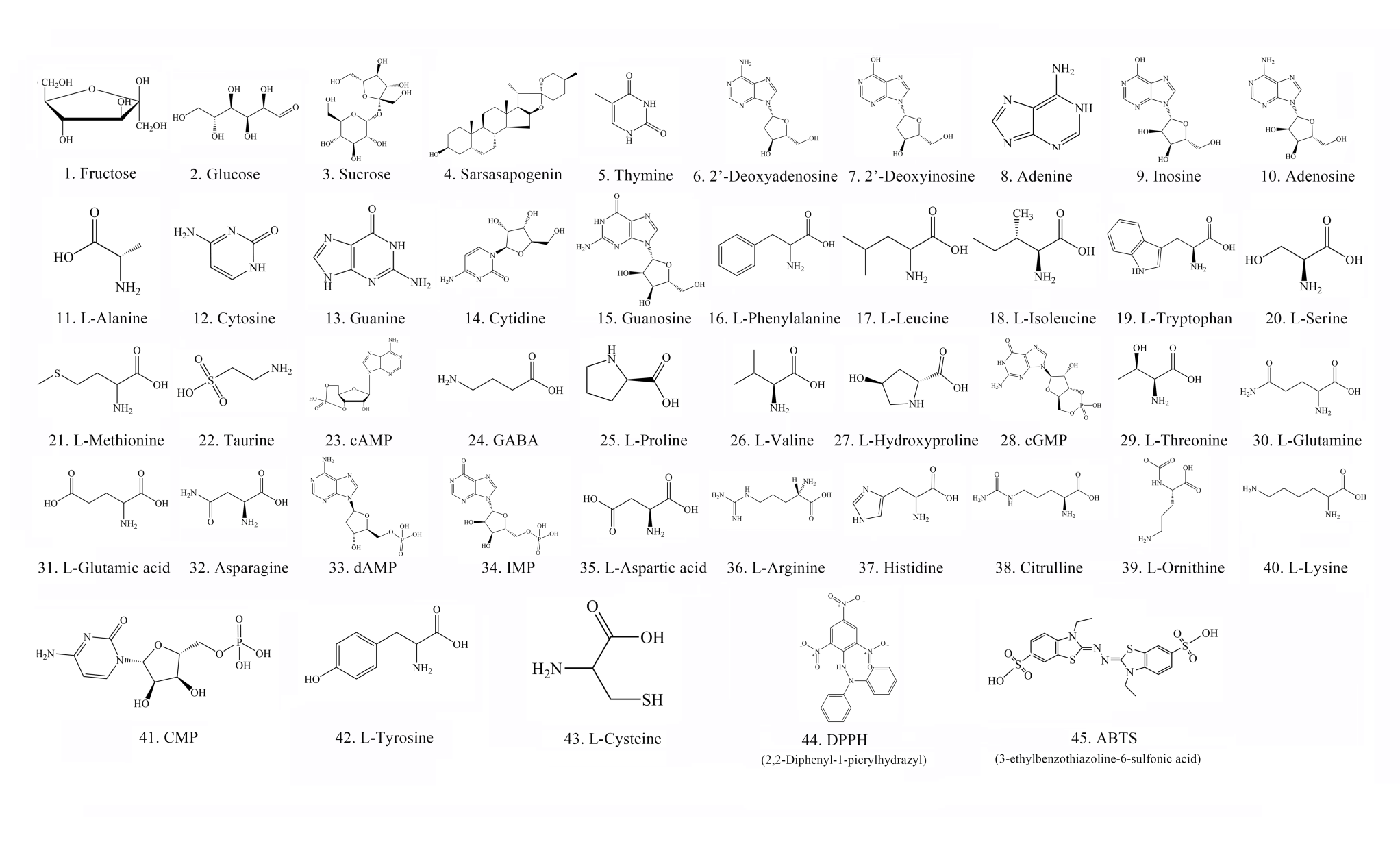


**Supplementary Figure 1.** Chemical structures of the standard compounds.


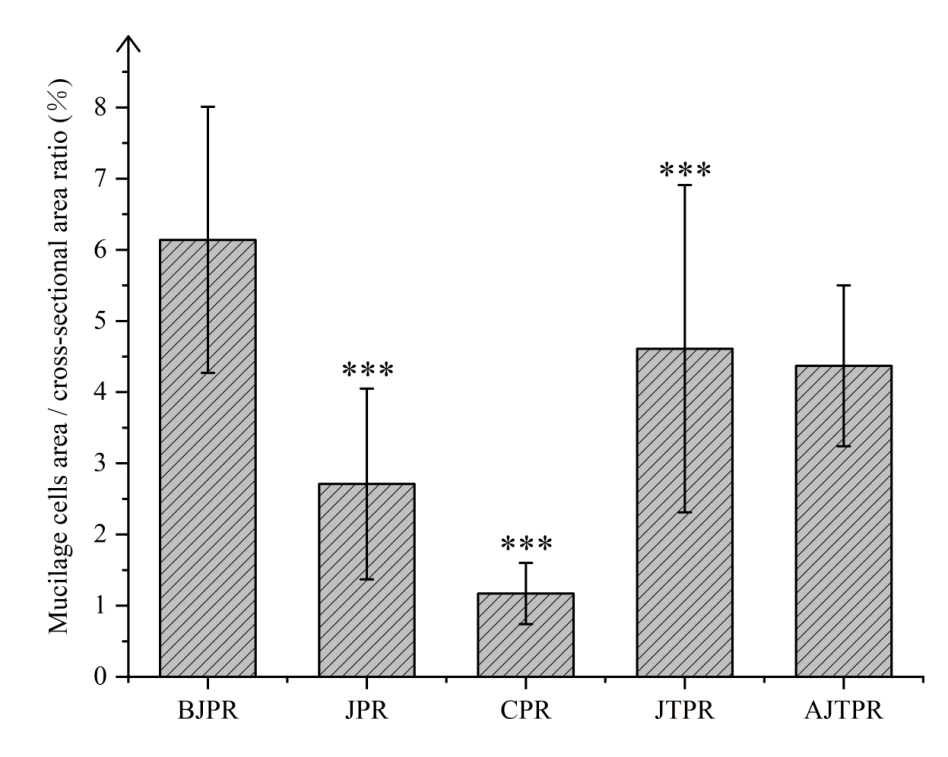


**Supplementary Figure 2.** The ratio of mucilage cell area to rhizome cross-sectional area (%).


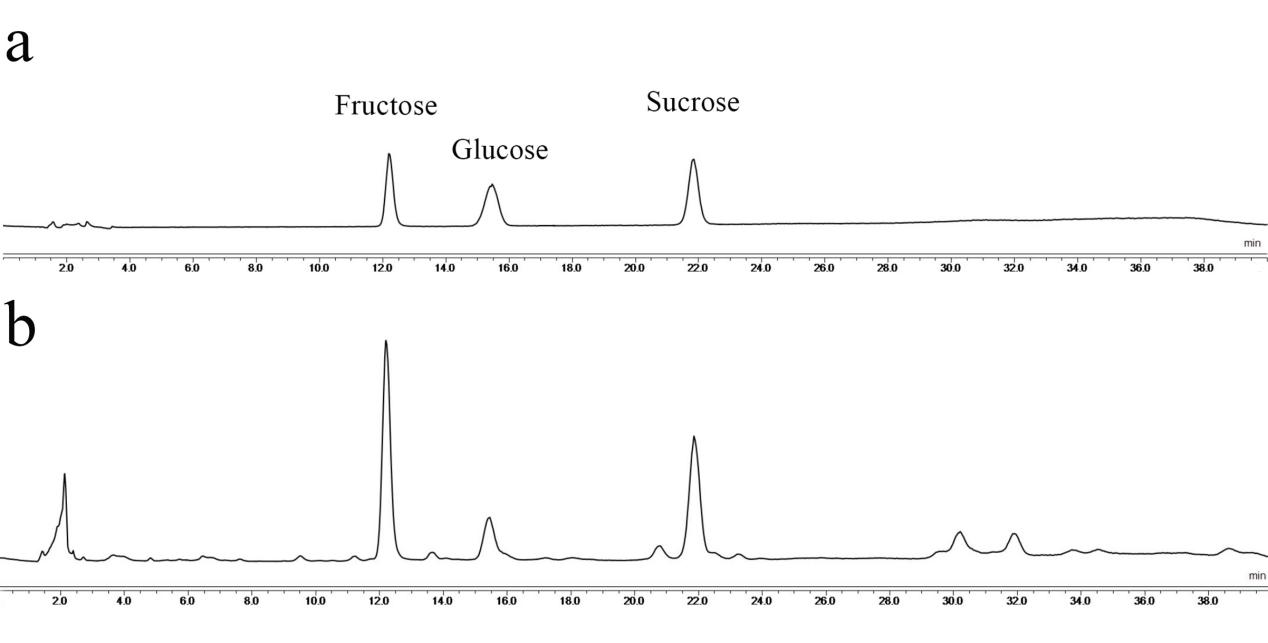


**Supplementary Figure 3.** High-pertypeance liquid chromatography coupled with charged aerosol detector (HPLC-CAD) chromatogram of mix standards (a) and samples (b) for 3 sugars of *Huangjing*.


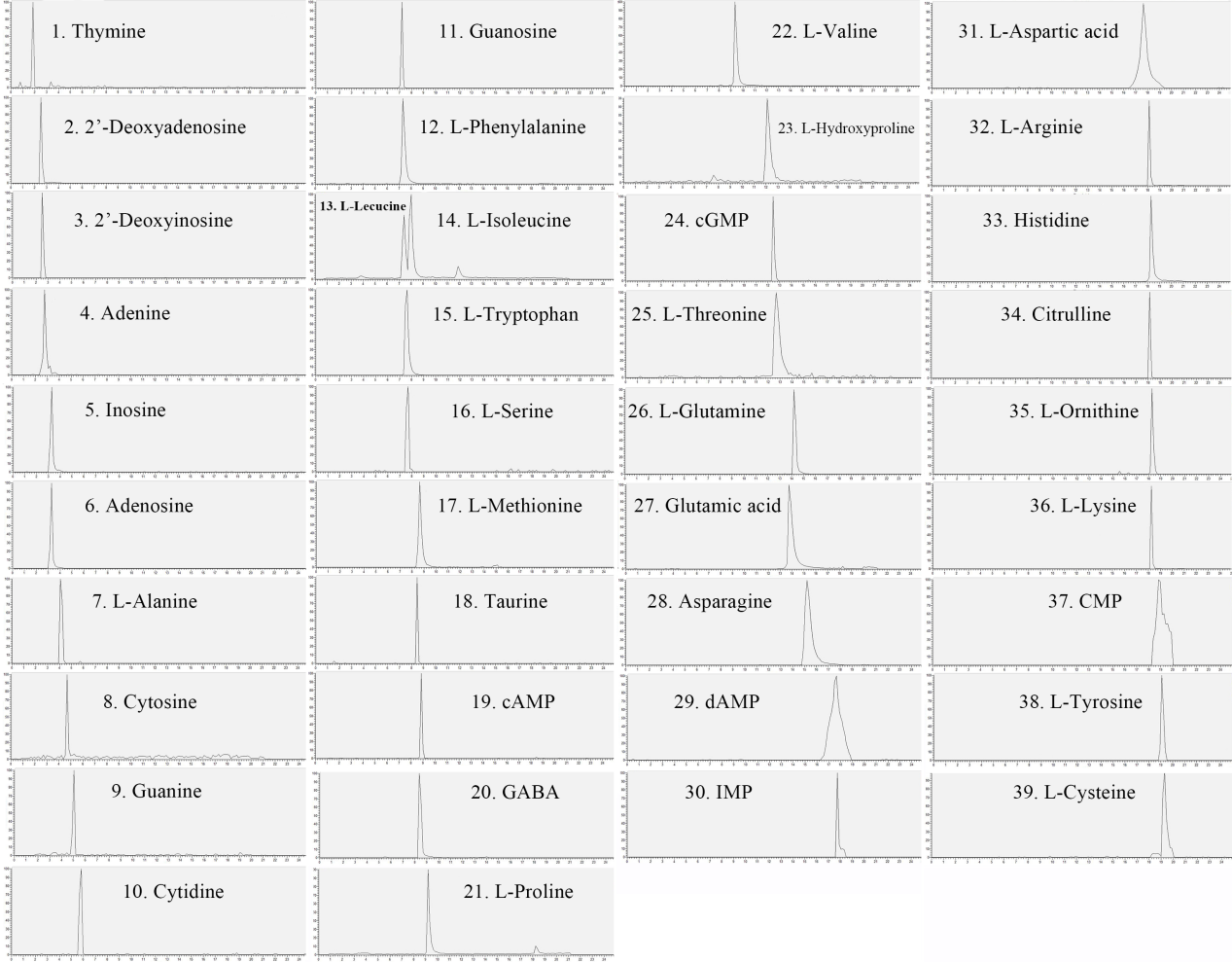


**Supplementary Figure 4.** Ultra-high pertypeance liquid chromatography - Orbitrap - tandem mass spectrometry (UHPLC - Orbitrap - MS/MS) chromatogram of 24 amino acids, 15 nucleosides and nucleobases.

| Species | Types | Collection Area | Collection Time | Sources | Latitude and Longitude | Note |
| --- | --- | --- | --- | --- | --- | --- |
| *P. cyrtonema* | *BaiJi*-type (BJPR) | Jinzhai, Luan,  Anhui | 2020.10.31 | Cultivated | 31°19’N  115°34’E | S1~S6 |
| *P. cyrtonema* | *Jiang*-type (JPR) | Jinzhai, Luan,  Anhui | 2020.10.31 | Cultivated | 31°19’N  115°34’E | S7~S18 |
| *P. cyrtonema* | *Cylinder*-type (CPR) | Jinzhai, Luan,  Anhui | 2020.11.07 | Cultivated | 31°19’N  115°34’E | S19~S24 |
| *P. sibiricum* | *JiTou*-type (JTPR) | Jinzhai, Luan,  Anhui | 2020.10.31 | Cultivated | 31°19’N  115°34’E | S25~S30 |
| *P. sibiricum* | *Atypical* “*JiTou*-type” (AJTPR) | Quanjiao, Chuzhou, Anhui | 2020.12.11 | Wild | 32°03’N  117°59’E | S31~S36 |

**Supplementary Table** **1.** The collecting places of *P. cyrtonema* and *P. sibiricum.*

**Supplementary Table** **2.** Detailed information of standards.

| **Reference compounds** | **Type** | **Manufacturer** |
| --- | --- | --- |
| [Asparagine](#/javascript:;) | Amino acid | Chengdu Herbpurify Co., Ltd |
| Citrulline | Amino acid | Chengdu Herbpurify Co., Ltd |
| GABA | Amino acid | Chengdu Lemeitian Pharmaceutical Technology Co., Ltd |
| Histidine | Amino acid | Chengdu Herbpurify Co., Ltd |
| L-Alanine | Amino acid | Chengdu Herbpurify Co., Ltd |
| L-Arginine | Amino acid | Chengdu Herbpurify Co., Ltd |
| L-Aspartic acid | Amino acid | Chengdu Herbpurify Co., Ltd |
| L-Cysteine | Amino acid | Chengdu Herbpurify Co., Ltd |
| L-Glutamic acid | Amino acid | Chengdu Herbpurify Co., Ltd |
| L-Glutamine | Amino acid | Chengdu Herbpurify Co., Ltd |
| L-Hydroxyproline | Amino acid | Chengdu Herbpurify Co., Ltd |
| L-Leucine | Amino acid | Chengdu Herbpurify Co., Ltd |
| Lysine | Amino acid | Chengdu Herbpurify Co., Ltd |
| L-Methionine | Amino acid | Chengdu Herbpurify Co., Ltd |
| L-Ornithine hydrochloride | Amino acid | Chengdu Herbpurify Co., Ltd |
| L-Phenylalanine | Amino acid | Chengdu Herbpurify Co., Ltd |
| L-Proline | Amino acid | Chengdu Herbpurify Co., Ltd |
| L-Serine | Amino acid | Chengdu Herbpurify Co., Ltd |
| L-Threonine | Amino acid | Chengdu Herbpurify Co., Ltd |
| L-Tryptophan | Amino acid | Chengdu Herbpurify Co., Ltd |
| L-Tyrosine | Amino acid | Chengdu Herbpurify Co., Ltd |
| L-Valine | Amino acid | Chengdu Herbpurify Co., Ltd |
| Taurine | Amino acid | Chengdu Herbpurify Co., Ltd |
| 2’-Deoxyadenosine | Nucleoside | Chengdu Lemeitian Pharmaceutical Technology Co., Ltd |
| 2’-Deoxyinosine | Nucleoside | Chengdu Lemeitian Pharmaceutical Technology Co., Ltd |
| Adenosine | Nucleoside | Chengdu Herbpurify Co., Ltd |
| cAMP | Nucleoside | Chengdu Lemeitian Pharmaceutical Technology Co., Ltd |
| Guanosine-3’,5’-cyclicmonophosphate sodiumsalt | Nucleoside | Chengdu Lemeitian Pharmaceutical Technology Co., Ltd |
| Cytidine 5'-monophosphate disodium salt | Nucleoside | Chengdu Lemeitian Pharmaceutical Technology Co., Ltd |
| Cytidine | Nucleoside | Chengdu Herbpurify Co., Ltd |
| dAMP | Nucleoside | Chengdu Lemeitian Pharmaceutical Technology Co., Ltd |
| Guanosine | Nucleoside | Chengdu Herbpurify Co., Ltd |
| Inosine 5'-monophosphate disodium salt | Nucleoside | Chengdu Lemeitian Pharmaceutical Technology Co., Ltd |
| Inosine | Nucleoside | Chengdu Herbpurify Co., Ltd |
| Adenine | Nucleobase | Chengdu Herbpurify Co., Ltd |
| Cytosine | Nucleobase | Chengdu Herbpurify Co., Ltd |
| Guanine | Nucleobase | Chengdu Herbpurify Co., Ltd |
| Thymine | Nucleobase | Chengdu Herbpurify Co., Ltd |
| D-Glucose | Saccharides | National Institutes for Food and Drug Control |
| Fructose | Saccharides | National Institutes for Food and Drug Control |
| Sucrose | Saccharides | National Institutes for Food and Drug Control |
| Sarsapogenin | Saponins | Chengdu Lemeitian Pharmaceutical Technology Co., Ltd |
| 3-Ethylbenzothiazoline-6-sulfonic acid (ABTS) | Antioxidant  reagent | Shanghai Macklin Biochemical Co., Ltd |
| 2,2-Diphenyl-1-picrylhydrazyl (DPPH) | Antioxidant  reagent | Shanghai yuanye Bio-Technology Co., Ltd |

|  | Fine end diameter/cm | Thick end diameter/cm | Stem scar diameter/cm | Internode length/cm | Scaly tooth height/cm | Scaly tooth width/cm | Annular spacing/cm | Branch angle of rhizomes/° |
| --- | --- | --- | --- | --- | --- | --- | --- | --- |
| BJPR | 1.36±0.41 | 1.83±0.30 | 0.88±0.31 | 3.53±0.66 | 2.67±0.5 | 1.01±0.22 | 0.28±0.16 | 57.95±15.56 |
| JPR | 2.5±0.67 | 3.15±0.79 | 1.60±0.46 | 6.41±2.04 | 2.23±0.62 | 1.25±0.38 | 0.99±0.29 | 64.38±14.36 |
| CPR | 1.76±0.50 | 2.21±0.56 | 0.85±0.36 | 4.63±2.53 | 1.45±0.49 | 0.55±0.07 | 0.67±0.27 | 95.56±7.26 |
| JTPR | 1.99±0.61 | 2.11±0.80 | 0.56±0.14 | 5.73±1.60 | 3.16±0.85 | 0.78±0.21 | 1.26±1.54 | 98.33±18.98 |
| AJTPR | 1.25±0.41 | 1.66±0.38 | 0.67±0.09 | 7.17±2.83 | 2.44±0.67 | 0.68±0.20 | 0.57±0.41 | 97.5±10.84 |

**Supplementary Table** **3.** Detailed data of the appearance features of the five types of *Huangjing.*

BJPR: “*BaiJi*-type” Polygonati rhizoma; JPR: “*Jiang*-type” Polygonati rhizoma; CPR: “*Cylinder*-type” Polygonati rhizoma; JTPR:“*JiTou*-type” Polygonati rhizoma; AJTPR: atypical “*JiTou*-type” Polygonati rhizoma.

**Supplementary Table** **4.** Regression curves, precision, repeatability, stability and recovery of three sugars.

|  | Calibration curves | tR  (min) | R2 | r | Linear Range | Precision | Repeatability | Stability | Recovery （n=6） | |
| --- | --- | --- | --- | --- | --- | --- | --- | --- | --- | --- |
|  |  |  |  |  | （mg/ml） | (RSD, n=6) | (RSD, n=6) |  | Mean | RSD |
| Fructose | y = 14.857x + 1.6846 | 12.3 | 0.998 | 0.9990 | 0.209 - 1.567 | 2.06% | 2.40% | 2.41% | 97.2% | 2.17% |
| Glucose | y = 15.880x + 0.5144 | 15.5 | 0.9989 | 0.9994 | 0.101 - 1.007 | 2.71% | 2.98% | 2.28% | 99.5% | 3.35% |
| Saccharose | y = 19.355x + 1.6630 | 21.9 | 0.999 | 0.9995 | 0.211 - 1.057 | 2.39% | 2.24% | 1.26% | 98.9% | 2.36% |

**Supplementary Table** 5**.** The ion mode and parameters for MRM of 24 amino acids, 15 nucleosides and nucleobases analytes.

|  | Analytes | Chemical  typeula | tR  (min) | Iron mode | Q1 | Q3 | CE  (N) |
| --- | --- | --- | --- | --- | --- | --- | --- |
| 1 | Thymine | C_5_H_6_N_2_O2 | 1.56 | [M + H]^+^ | 127.0502 | 110.02388 | 30 |
| 2 | 2’-Deoxyadenosine | C_10_H_13_N_5_O_3_ | 2.39 | [M + H]^+^ | 252.1091 | 136.06206 | 22 |
| 3 | 2’-Deoxyinosine | C_10_H_12_N_4_O_4_ | 2.40 | [M + H]^+^ | 253.0931 | 136.06210 | 22 |
| 4 | Adenine | C_5_H_5_N_5_ | 2.66 | [M + H]^+^ | 136.0618 | 94.03999 | 40 |
| 5 | Inosine | C_10_H_12_N_4_O_5_ | 2.95 | [M + H]^+^ | 269.0880 | 136.06210 | 22 |
| 6 | Adenosine | C_10_H_13_N_5_O_4_ | 2.96 | [M + H]^+^ | 268.1040 | 136.06210 | 22 |
| 7 | L-Alanine | C_3_H_7_NO_2_ | 3.82 | [M + H]^+^ | 90.0550 | 72.08099 | 30 |
| 8 | Cytosine | C_4_H_5_N_3_O | 4.02 | [M + H]^+^ | 112.0505 | 69.04498 | 32 |
| 9 | Guanine | C_5_H_5_N_5_O | 4.85 | [M + H]^+^ | 152.0567 | 110.03513 | 30 |
| 10 | Cytidine | C_9_H_13_N_3_O_5_ | 5.54 | [M + H]^+^ | 244.0928 | 112.05086 | 22 |
| 11 | Guanosine | C_10_H_13_N_5_O_5_ | 6.90 | [M + H]^+^ | 284.0989 | 152.05698 | 22 |
| 12 | L-Phenylalanine | C_9_H_11_NO_2_ | 7.03 | [M + H]^+^ | 166.0863 | 120.08105 | 22 |
| 13 | L-Leucine & L-Isoleucine | C_6_H_13_NO_2_ | 7.05 | [M + H]^+^ | 132.1019 | 86.09665 | 22 |
| 14 | L-Tryptophan | C_11_H_12_N_2_O_2_ | 7.31 | [M + H]^+^ | 205.0972 | 188.07101 | 22 |
| 15 | L-Serine | C_3_H_7_NO_3_ | 7.63 | [M + H]^+^ | 106.0499 | 70.06540 | 22 |
| 16 | L-Methionine | C_5_H_11_O_2_NS | 8.27 | [M + H]^+^ | 150.0583 | 104.05309 | 22 |
| 17 | Taurine | C_2_H_7_NO_3_S | 8.54 | [M + H]^+^ | 126.0219 | 108.01157 | 25 |
| 18 | cAMP | C_10_H_12_N_5_O_6_P | 8.71 | [M + H]^+^ | 330.0598 | 136.06210 | 32 |
| 19 | GABA | C_4_H_9_NO_2_ | 8.93 | [M + H]^+^ | 104.0706 | 87.04429 | 22 |
| 20 | L-Proline | C_5_H_9_NO_2_ | 9.08 | [M + H]^+^ | 116.0706 | 70.06540 | 22 |
| 21 | L-Valine | C_5_H_11_NO_2_ | 9.09 | [M + H]^+^ | 118.0863 | 72.08102 | 22 |
| 22 | L-Hydroxyproline | C_5_H_9_NO_3_ | 11.66 | [M + H]^+^ | 132.0655 | 86.06020 | 25 |
| 23 | cGMP | C_10_H_12_N_5_O_7_P | 12.23 | [M + H]^+^ | 346.0547 | 152.05682 | 35 |
| 24 | L-Threonine | C_4_H_9_NO_3_ | 12.50 | [M + H]^+^ | 120.0655 | 74.06023 | 22 |
| 25 | L-Glutamine | C_5_H_10_N_2_O_3_ | 14.26 | [M + H]^+^ | 147.0764 | 84.04458 | 22 |
| 26 | L-Glutamic acid | C_5_H_9_NO_4_ | 14.37 | [M + H]^+^ | 148.0604 | 84.04459 | 50 |
| 27 | [Asparagine](#javascript:;) | C_4_H_8_N_2_O_3_ | 14.92 | [M + H]^+^ | 133.0608 | 87.05551 | 25 |
| 28 | dAMP | C_10_H_14_N_5_O_6_P | 17.51 | [M + H]^+^ | 332.0754 | 136.06201 | 40 |
| 29 | Inosine -5- monophosphate | C_10_H_13_N_4_O_8_P | 17.82 | [M + H]^+^ | 349.0544 | 137.06534 | 35 |
| 30 | [L-Aspartic acid](#javascript:;) | C_4_H_7_NO_4_ | 17.95 | [M + H]^+^ | 134.0448 | 74.02384 | 22 |
| 31 | L-Arginine | C_6_H_14_N_4_O_2_ | 18.06 | [M + H]^+^ | 175.1190 | 70.06538 | 33 |
| 32 | Histidine | C_6_H_9_N_3_O_2_ | 18.07 | [M + H]^+^ | 156.0768 | 110.07154 | 22 |
| 33 | Citrulline | C_6_H_13_N_3_O_3_ | 18.14 | [M + H]^+^ | 176.1030 | 113.09044 | 40 |
| 34 | L-Ornithine | C_5_H_12_N_2_O_2_ | 18.21 | [M + H]^+^ | 133.0972 | 70.06538 | 35 |
| 35 | Lysine | C_6_H_14_N_2_O_2_ | 18.21 | [M + H]^+^ | 147.1128 | 84.08098 | 43 |
| 36 | Cytidine -5- monophosphate | C_9_H_14_N_3_O_8_P | 18.42 | [M + H]^+^ | 324.0591 | 112.05071 | 35 |
| 37 | L-Tyrosine | C_9_H_11_NO_3_ | 18.70 | [M + H]^+^ | 182.0812 | 136.00133 | 22 |
| 38 | L-Cysteine | C_3_H_7_NO_2_S | 18.98 | [M + H]^+^ | 122.0270 | 79.01807 | 25 |

**Supplementary Table** **6.** Regression curves, Linear range, LOD, LOQ of 24 amino acids, 15 nucleosides and nucleobases analytes.

| Analytes | Regression equation | R^2^ | r | Linear range (ng/mL) | | LOD(ng/mL) | LOQ(ng/mL) |
| --- | --- | --- | --- | --- | --- | --- | --- |
| L-Cysteine | y = 4826x + 161851 | 0.9989 | 0.9994 | 5.00 | 1000 | 1.60 | 4.50 |
| L-Phenylalanine | y = 49546x -1521474 | 0.9981 | 0.9990 | 200 | 3700 | 0.80 | 3.00 |
| L-Alanine | y = 1214x + 82338 | 0.9991 | 0.9995 | 100 | 3000 | 10.0 | 35.0 |
| L-Glutamine | y = 4012x + 65960 | 0.9997 | 0.9998 | 30.0 | 26000 | 5.50 | 18.0 |
| L-Glutamic acid | y = 7988x -3155803 | 0.9985 | 0.9992 | 1000 | 14000 | 6.00 | 20.0 |
| L-Methionine | y = 4238x + 207039 | 0.9987 | 0.9993 | 30.0 | 3000 | 5.00 | 16.0 |
| L-Arginine | y = 2247x + 5432971 | 0.9997 | 0.9998 | 10000 | 400000 | 0.25 | 1.00 |
| Lysine | y = 2718x + 320420 | 0.9982 | 0.9991 | 500 | 20000 | 0.30 | 1.00 |
| L-Tyrosine | y = 317x + 1371 | 0.9988 | 0.9994 | 10.0 | 1000 | 3.30 | 8.40 |
| L-Leucine | y = 27599x + 1965207 | 0.9992 | 0.9996 | 500 | 8000 | 0.36 | 1.20 |
| L-Ornithine | y = 3278x - 851658 | 0.9981 | 0.9990 | 200 | 4000 | 5.00 | 17.0 |
| L-Proline | y = 42893x + 122240702 | 0.9964 | 0.9982 | 2000 | 30000 | 0.30 | 1.00 |
| L-Hydroxyproline | y = 15603x - 329405 | 0.9988 | 0.9994 | 120 | 1500 | 0.75 | 2.50 |
| L-Tryptophan | y = 7702x - 965006 | 0.999 | 0.9995 | 40.0 | 8000 | 7.50 | 20.0 |
| L-Serine | y = 1368x - 367150 | 0.9994 | 0.9997 | 10000 | 120000 | 28.0 | 95.0 |
| L-Threonine | y = 10679x - 67716250 | 0.9969 | 0.9984 | 8000 | 80000 | 4.60 | 15.0 |
| L-Aspartic acid | y = 29.8x + 152751 | 0.9993 | 0.9996 | 3000 | 600000 | 3.00 | 10.0 |
| L-Valine | y = 16411x - 5130648 | 0.9880 | 0.9940 | 800 | 5020 | 1.20 | 4.00 |
| Citrulline | y = 21.8x + 383043 | 0.9992 | 0.9996 | 10000 | 500000 | 35.0 | 115 |
| Taurine | y = 12421x + 3546.3 | 0.9849 | 0.9924 | 0.10 | 50 | 0.05 | 0.10 |
| GABA | y = 10463 x + 2440269 | 0.9981 | 0.9990 | 320 | 5000 | 2.50 | 8.50 |
| [Asparagine](#/javascript:;) | y = 714x + 538157 | 0.9986 | 0.9993 | 300 | 30000 | 18.0 | 60.0 |
| Histidine | y = 28126 x - 4299217 | 0.9981 | 0.9990 | 100 | 7000 | 0.25 | 1.00 |
| 2’-Deoxyinosine | y = 19360x - 1174 | 0.9985 | 0.9992 | 2.00 | 200 | 0.40 | 1.50 |
| 2’-Deoxyadenosine | y = 654911x - 281744 | 0.9827 | 0.9913 | 1.00 | 20 | 0.24 | 0.90 |
| Cytidine | y = 6227x - 4413 | 0.9992 | 0.9996 | 5.00 | 380 | 0.20 | 0.70 |
| Inosine | y = 11324x - 434123 | 0.9991 | 0.9995 | 80.0 | 550 | 7.50 | 18.6 |
| Guanosine | y = 6070x + 230163 | 0.998 | 0.9990 | 10.0 | 2000 | 1.00 | 3.00 |
| Adenosine | y = 255100x - 901700 | 0.9961 | 0.9980 | 50.0 | 400 | 1.00 | 3.50 |
| IMP | y = 73.0x - 4049 | 0.9981 | 0.9990 | 500 | 5000 | 0.40 | 1.50 |
| CMP | y = 1887x - 94492 | 0.9952 | 0.9976 | 40.0 | 570 | 4.00 | 15.0 |
| cGMP | y = 20748x - 55061 | 0.9994 | 0.9997 | 3.00 | 200 | 0.50 | 2.00 |
| cAMP | y = 9037x + 80628 | 0.9991 | 0.9995 | 10.0 | 600 | 1.20 | 4.00 |
| dAMP | y = 898x + 3626 | 0.9984 | 0.9992 | 1.00 | 160 | 0.10 | 0.40 |
| Cytosine | y = 11680x - 6156 | 0.9987 | 0.9993 | 1.00 | 100 | 0.05 | 0.20 |
| Guanine | y = 2850x - 1962 | 0.9961 | 0.9980 | 2.50 | 50 | 0.10 | 0.40 |
| Adenine | y = 7682x - 7818 | 0.9976 | 0.9988 | 1.00 | 35 | 0.25 | 1.00 |
| Thymine | y = 3270x - 361 | 0.9982 | 0.9991 | 1.00 | 50 | 0.20 | 0.50 |

**Supplementary Table** **7.** Precision, repeatability, stability and recovery of 24 amino acids, 15 nucleosides and nucleobases analytes.

|  | Precision  (RSD,%, n=6) | Repeatability  (RSD,%, n=6) | Stability  (RSD,%) | Recovery （n=6） | |
| --- | --- | --- | --- | --- | --- |
|  |  |  |  | Mean (%) | RSD (%) |
| L-Cysteine | 1.91 | 2.54 | 1.65 | 96.8 | 4.78 |
| L-Phenylalanine | 2.66 | 2.92 | 3.04 | 97.5 | 1.32 |
| L-Alanine | 3.48 | 4.97 | 2.55 | 93.1 | 2.57 |
| L-Glutamine | 4.60 | 3.00 | 4.86 | 103.5 | 1.69 |
| L-Glutamic acid | 2.33 | 3.02 | 4.06 | 107.2 | 1.36 |
| L-Methionine | 1.69 | 1.64 | 3.64 | 90.6 | 3.25 |
| L-Arginine | 2.91 | 2.58 | 2.60 | 97.7 | 4.68 |
| Lysine | 3.80 | 2.34 | 3.78 | 90.3 | 3.95 |
| L-Tyrosine | 4.41 | 5.66 | 2.59 | 106.4 | 3.27 |
| L-Leucine | 3.54 | 2.23 | 3.85 | 104.3 | 3.22 |
| L-Ornithine | 1.30 | 4.20 | 4.44 | 104.1 | 3.75 |
| L-Proline | 3.86 | 0.98 | 2.11 | 94.3 | 2.71 |
| L-Hydroxyproline | 1.97 | 2.99 | 5.28 | 96.4 | 3.45 |
| L-Tryptophan | 3.91 | 1.63 | 4.80 | 91.2 | 2.25 |
| L-Serine | 3.60 | 4.55 | 4.61 | 103.4 | 3.46 |
| L-Threonine | 1.62 | 2.51 | 5.85 | 105.3 | 1.24 |
| L-Aspartic acid | 4.96 | 5.83 | 4.87 | 96.4 | 2.96 |
| L-Valine | 1.34 | 3.44 | 4.59 | 94.1 | 1.98 |
| Citrulline | 2.16 | 1.42 | 4.52 | 106.6 | 4.38 |
| Taurine | 3.93 | 1.89 | 7.14 | 92.5 | 1.63 |
| GABA | 2.25 | 1.85 | 2.86 | 93.4 | 3.89 |
| [Asparagine](file:///C:\Downloads\Application\8.9.6.0\resultui\html\index.html#/javascript:;) | 2.91 | 3.04 | 5.14 | 89.7 | 2.31 |
| Histidine | 3.71 | 3.63 | 5.22 | 95.6 | 3.08 |
| 2’-Deoxyinosine | 5.92 | 2.64 | 5.94 | 85.4 | 4.68 |
| 2’-Deoxyadenosine | 5.78 | 1.92 | 5.50 | 88.3 | 4.96 |
| Cytidine | 2.90 | 3.08 | 2.69 | 90.6 | 3.71 |
| Inosine | 1.87 | 2.52 | 2.55 | 93.4 | 3.45 |
| Guanosine | 4.35 | 4.46 | 3.88 | 99.6 | 4.22 |
| Adenosine | 1.82 | 1.30 | 2.13 | 87.6 | 2.87 |
| IMP | 4.59 | 3.22 | 6.34 | 96.5 | 2.25 |
| CMP | 4.69 | 5.58 | 4.07 | 91.0 | 3.97 |
| cGMP | 4.64 | 5.43 | 6.57 | 86.2 | 5.34 |
| cAMP | 4.24 | 3.97 | 4.18 | 90.3 | 4.58 |
| dAMP | 3.02 | 3.70 | 5.56 | 95.2 | 3.55 |
| Cytosine | 3.95 | 4.80 | 2.10 | 108.6 | 4.76 |
| Guanine | 3.68 | 3.22 | 5.37 | 93.5 | 5.15 |
| Adenine | 2.77 | 3.55 | 4.74 | 96.3 | 3.32 |
| Thymine | 2.40 | 5.08 | 5.68 | 106.7 | 4.59 |

**Supplementary Table** **8.** The comprehensive score and ranking of *Huangjing* from different types by PCA.

| Variety | Principal component scores | | | | Composite score | Ranking |
| --- | --- | --- | --- | --- | --- | --- |
|  | F1 | F2 | F3 | F4 |  |  |
| BJPR | 2.952 | -1.038 | 3.031 | 0.716 | 1.474 | 1 |
| JPR | -0.681 | -1.214 | -0.090 | -1.201 | -0.601 | 4 |
| CPR | -4.066 | -0.711 | -0.480 | 1.036 | -1.624 | 5 |
| JTPR | 3.330 | 0.365 | -2.951 | 0.436 | 0.774 | 2 |
| AJTPR | -0.855 | 3.812 | 0.581 | 0.213 | 0.577 | 3 |

BJPR: “*BaiJi*-type” Polygonati rhizoma; JPR: “*Jiang*-type” Polygonati rhizoma; CPR: “*Cylinder*-type” Polygonati rhizoma; JTPR:“*JiTou*-type” Polygonati rhizoma; AJTPR: atypical “*JiTou*-type” Polygonati rhizoma.
