# Supplementary figures and images for: An Evaluation of Traits, Nutritional, and Medicinal Component Quality of Polygonatum cyrtonema Hua and P. sibiricum Red
Source: Front Plant Sci. 2022 Apr 18;13:891775. doi: 10.3389/fpls.2022.891775 (PMC9062581; doi:10.3389/fpls.2022.891775)

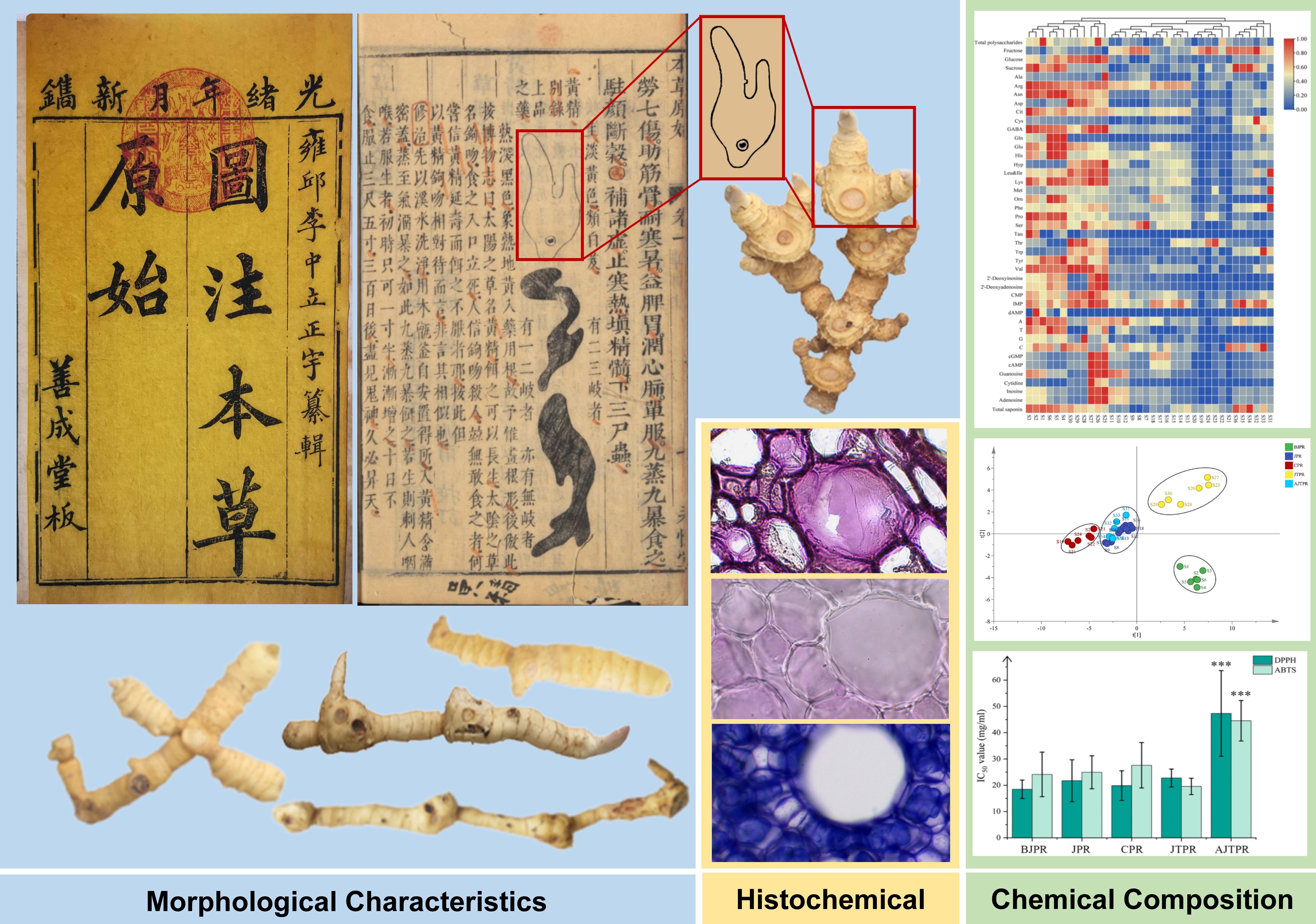

Supplement: Supplementary file 2 [file Image_1.jpg]
